# Global Trends and Research Hotspots in Natural Products for Hyperuricemia: A Bibliometric Analysis (2004–2026)

**DOI:** 10.3390/ph19081286

**Published:** 2026-08-14

**Authors:** Shuang Pan, Yumo Li, Zhi Lin, Zhe Lin, He Lin, Ying Lv

**Affiliations:** 1 College of Pharmacy, Changchun University of Chinese Medicine, Changchun 130117, China; 2 School of Pharmaceutical Sciences, Zhejiang Chinese Medical University, Hangzhou 311402, China; 3 College of Basic Medicine, Changchun University of Chinese Medicine, Changchun 130117, China

**Keywords:** bibliometric analysis, NPs, HUA, Bibliometrix, VOSviewer, CiteSpace

## Abstract

Hyperuricemia (HUA) is a key pathological basis for gout and uric acid (UA) nephropathy, and its increasing global prevalence presents a substantial public health challenge. Natural products (NPs) have emerged as a research hotspot due to their potential to reduce UA and favourable safety profile; however, comprehensive bibliometric analyses in this area remain limited. In this study, the English literature on NPs with anti-HUA activity published between 2004 and 2026 was retrieved from the Web of Science, Scopus, and PubMed, and analyzed using Bibliometrix (R package), VOSviewer, and CiteSpace. A total of 581 articles were identified, indicating a rapid increase in publication output across 53 countries, 870 institutions, 234 journals, and 3421 authors. China was found to lead the field, with Guangzhou University of Traditional Chinese Medicine contributing the highest number of publications. The *Journal of Ethnopharmacology* ranked first in publication volume, and Kong Ling-Dong emerged as the most prolific author. Major research foci included xanthine oxidase (XOD), UA metabolism, oxidative stress, inflammatory regulation, gut microbiota, and molecular docking. *Perilla frutescens* leaves and *Terminalia chebula*, together with six principal categories of bioactive compounds, demonstrated promising therapeutic potential. Overall, this study elucidates the developmental trends and research frontiers in the field, providing a valuable reference for future investigations.

## 1. Introduction

Hyperuricemia (HUA) is a metabolic disorder characterized by elevated serum uric acid (SUA) levels [1]. UA is the final product of endogenous purine metabolism in humans, and its serum homeostasis primarily depends on the balance between hepatic production and renal or intestinal excretion [2]. Clinically, HUA is defined as SUA levels of ≥7.0 mg/dL (420.0 μmol/L) in males or ≥6.0 mg/dL (360.0 μmol/L) in females [3]. When UA excretion is impaired or its production is excessive, elevated SUA levels can result in HUA [3]. This condition is influenced by multiple factors, including obesity [4], hypertension [5], alcohol consumption [6], and a high-purine diet [7]. HUA is further associated with complications such as gout [8], hypertension [9], kidney disease [10], and cardiovascular disease [11]. Globally, HUA is the second-most prevalent metabolic disorder after diabetes, posing a significant threat to public health [12,13]. Studies have reported that the prevalence of HUA reaches 26.6% in South Korea [14], approximately 20% in the USA [15], and 16.4% in China [16].

Currently, the primary pharmacological agents for the clinical management of HUA include xanthine oxidase (XOD) inhibitors [17], urate transporter 1 (URAT1) inhibitors [18], and recombinant urate oxidase [19]. However, these agents have several limitations, including prolonged dependence on therapy, severe adverse events such as hypersensitivity responses [20], cardiovascular complications [21], hepatotoxicity [22], interactions between drugs, and hemolytic risk [19], along with substantial treatment costs and immunogenicity concerns associated with biologic agents. Consequently, clinical management of HUA remains challenging, and the development of safe, cost-effective, and sustainable novel therapies is a major unresolved global medical priority.

Compared with chemically synthesized drugs, therapies based on natural products (NPs) have attracted increasing research attention, owing to their potential for low toxicity, multiple target modulation properties, and cost effectiveness [23,24]. In recent years, NPs such as *Chrysanthemum morifolium* [25], *Plantago ovata* [26], *Apium graveolens* [27], *Cichorium intybus* [28], and *Camellia sinensis* [29] have emerged as important sources for the development of UA-lowering agents. These compounds exert therapeutic effects by regulating key pathways in UA metabolism, including inhibition of XOD, downregulation of URAT1 expression, activation of ATP-binding cassette transporter G2 (ABCG2)-mediated efflux, and modulation of inflammatory pathways. Collectively, these mechanisms can lower SUA levels, attenuate renal inflammatory injury, and thereby improve therapeutic safety and patient adherence. However, most evidence supporting the efficacy of NPs against HUA remains limited to in vitro experiments and animal models. With the rapid expansion of research in this field, systematically analyzing core researchers, institutional networks, journal contributions, and keyword trends is essential for understanding the evolution of the discipline and promoting clinical translation.

In response, the present study employs a bibliometric analysis. Compared with traditional literature review approaches, bibliometrics is a quantitative research method designed to systematically measure and analyze the multidimensional characteristics of literature, such as keywords, authors, institutions, and citation networks, through the integration of mathematics, statistics, and computer science techniques. This method objectively reveals the knowledge structure, developmental trends, core themes, and research frontiers within a specific field [30]. Its core principle lies in using data to quantify bibliographic information and applying visualization tools to present complex knowledge relationships, thereby enabling researchers to rapidly grasp the essence of a field and mitigate the subjective bias inherent in traditional reviews [31,32]. In addition, R is a widely used open-source programming language, integrating extensive bibliometric analysis packages that support multidimensional and comprehensive assessments of literature data, including keyword co-occurrence, author collaboration networks, and citation trend analyses. The language is characterized by ease of use, comprehensive functionality, and high analytical efficiency. It has been widely applied across diverse research domains, including medicine, biology, and management [33,34,35,36].

Previous bibliometric studies have examined related areas, including traditional Chinese medicine (TCM), HUA, and gout. A PubMed-based study analyzed TCM publications from 1995 to 2014 and characterized their global publication patterns [37]. A Web of Science study summarized the major research clusters and developmental directions in HUA research between 1980 and 2019 [38]. Another study based on the Web of Science Core Collection (WoSCC) examined trends in gout research from 2012 to 2021 and identified pathological mechanisms, gut microbiota, the NLRP3 inflammasome, XOD, and urate transporters as important research themes [39]. In addition, a WoSCC-based study examined publications on NPs in HUA research up to 2021. Flavonoids, antioxidant activity, and XOD were among the topics addressed [40].

Most of these studies relied on a single database, and the latest publication year covered was 2021. The relationships between different NPs categories and their specific mechanisms of action have not been systematically integrated. In the present study, records were retrieved from Web of Science, Scopus, and PubMed, and the study period was extended to 1 April 2026. Following screening, 581 publications were included. Bibliometric analysis was further integrated with a review of six major NPs categories and their potential mechanisms in HUA.

## 2. Methods

### 2.1. Data Sources and Search Strategy

On 1 April 2026, simultaneous searches were conducted in three databases: the WoSCC, PubMed, and Scopus. The search period was defined as 1 January 2004 to 1 April 2026. Preliminary scoping searches indicated that only a small number of studies had been published before 2004 and that these records did not form a stable or comparable literature set. Sustained and systematic research activity became evident from 2004 onward. The cutoff date was fixed at 1 April 2026 to reduce temporal variation caused by ongoing database updates.

The search strategy comprised two conceptual blocks: terms related to NPs and traditional medicine, and terms related to HUA and UA metabolism. Search terms were selected based on commonly used terminology in related studies and were expanded with PubMed Medical Subject Headings (MeSH). Synonyms and spelling variants were also included to improve search sensitivity. The two blocks were combined using the Boolean operator AND. Searches were performed in the Topic field in WoSCC, the Title/Abstract and MeSH Terms fields in PubMed, and the TITLE-ABS-KEY fields in Scopus.

The screening workflow was adapted to the native filtering functions of each database. In the present workflow, PubMed and Scopus were not filtered by an exact day through their sidebar interfaces. Accordingly, the publication period, document type, and language criteria were embedded in the search strings for these two databases and applied sequentially in four cumulative steps. In WoSCC, the date range was specified using the built-in Publication Date sidebar, which supports the YYYY-MM-DD format, whereas document type (Article and Review) and language (English) were embedded in the search string. The complete search strings and database-specific screening procedures are provided in the Supplementary Methods.

### 2.2. Data Preprocessing

All records retrieved from the three databases were exported and merged into a single dataset for screening. Duplicate records were identified and removed using EndNote (version 21; Clarivate, London, UK). Author, publication year, article title, and document type were used as matching fields, followed by manual verification.

English-language original research articles and reviews relevant to NPs-based treatment of HUA were included. These document types were selected because they generally represent complete, peer-reviewed scholarly outputs suitable for bibliometric analysis.

Records were excluded if they were irrelevant to the research topic, published in a language other than English, or belonged to document types other than original research articles and reviews. The excluded types included conference abstracts, editorials, letters, conference proceedings, books, book chapters, and retracted publications.

Records with incomplete metadata were retained in the overall dataset when core bibliographic information, including the title, authors, source, and publication year, were available. Missing fields, including author keywords, abstracts, and affiliations, were not imputed. Such records were excluded only from analyses that required the corresponding fields.

**Figure 1:**
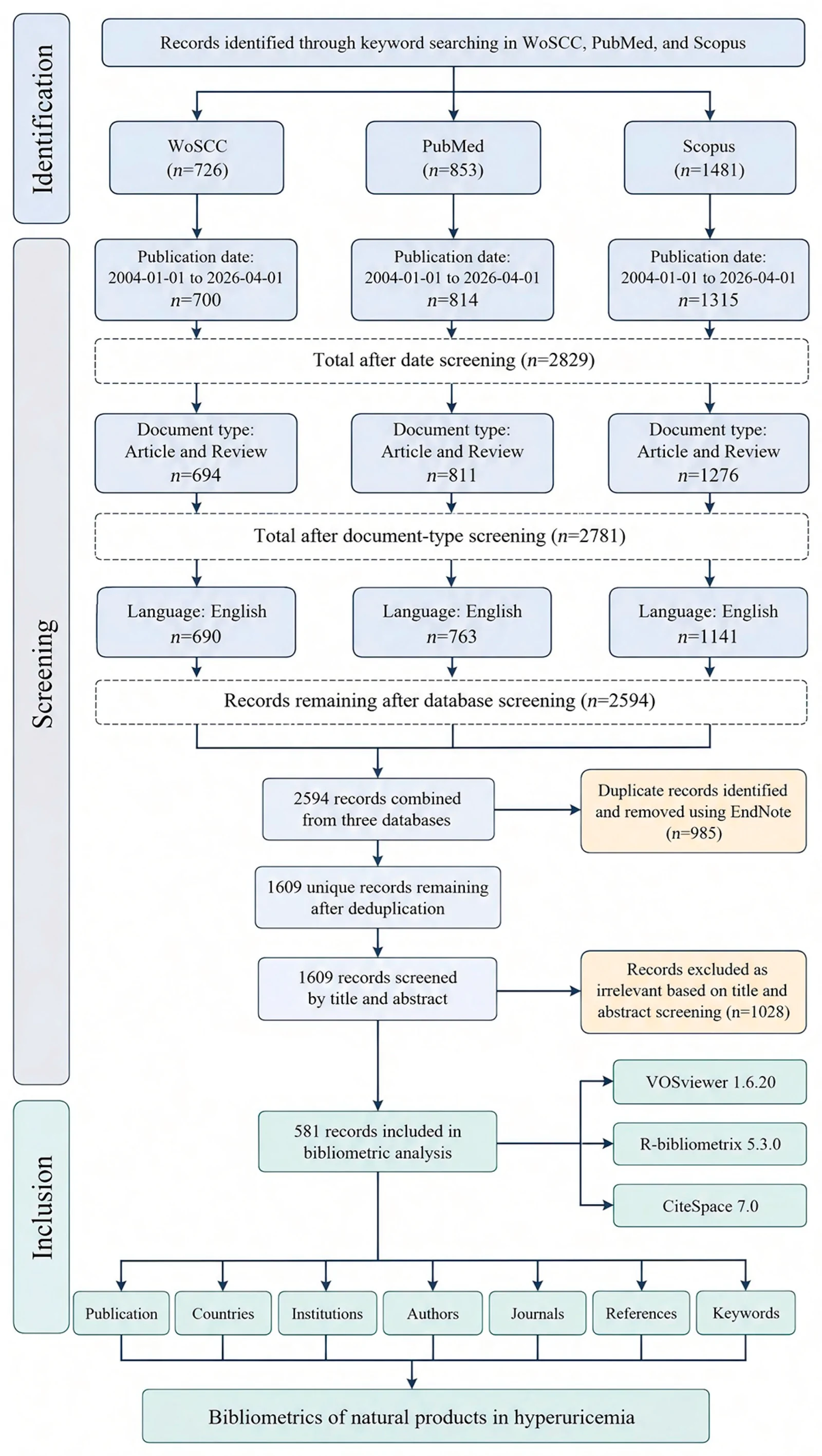
Literature identification, screening, and inclusion flowchart. (Because Scopus does not support publication-date filtering at the month-and-day level, the publication year range of 2004–2026 was applied. The database searches were conducted on 1 April 2026).

The 581 included publications were subsequently imported into Bibliometrix R package (version 5.3.0; Massimo Aria and Corrado Cuccurullo, University of Naples Federico II, Naples, Italy), VOSviewer (version 1.6.20; Nees Jan van Eck and Ludo Waltman, Leiden University, Leiden, the Netherlands), and CiteSpace (version 7.0; Chaomei Chen, Drexel University, Philadelphia, PA, USA) for bibliometric and visualization analyses. The overall study workflow is shown in Figure 1.

### 2.3. Data Analysis

This study employed bibliometric tools, including the Bibliometrix R package, VOSviewer, and CiteSpace, to visualize and analyze the results of the bibliometric analysis. To ensure comprehensive coverage of all analytical dimensions, appropriate software packages were selected based on their specific capabilities. Specifically, R (version 4.6.0; R Foundation for Statistical Computing, Vienna, Austria) with the Bibliometrix package (version 5.3.0), CiteSpace (version 7.0), and VOSviewer (version 1.6.20) were used to perform visual analyses of the selected literature. These tools facilitated in-depth assessments of publications, countries, institutions, journals, authors, and keywords. The R package Bibliometrix [41] was employed to calculate the frequencies of journals, institutions, and countries, with the top ten results in each category reported. The ggplot2 package (version 4.0.2; Posit PBC, Boston, MA, USA) was applied to enhance the visual presentation of the outputs.

VOSviewer, developed by Nees Jan van Eck and Ludo Waltman at Leiden University [42], was employed to generate co-occurrence networks for authors, countries, institutions, journals, and keywords, in which node size represented occurrence or citation frequency. The minimum thresholds were set as follows: one publication for country collaboration analysis, five occurrences for institutional collaboration analysis, five publications for author collaboration analysis, 30 citations for co-cited author analysis, and 120 citations for co-cited journal analysis.

To assess the research activity and influence of different countries and regions in NPs-based HUA research, country-level analyses were conducted using publication output, total citations, average citations per paper (AC/P), and the H-index. International collaboration patterns were visualized using collaboration networks. Publications were also classified as single-country publications (SCP) or multiple-country publications (MCP) to distinguish independent research from cross-border collaborative output.

Keyword analysis was based on all keywords. Before analysis, synonymous terms were standardized by harmonizing special-character variants, merging singular and plural forms, and standardizing British and American spelling. The minimum occurrence threshold for keyword analysis was set at >10.

CiteSpace, developed by Professor Chaomei Chen [43], was utilized for the analysis of keyword burst frequency and the visualization of clustering timelines. During the process, the sliced network was pruned, merged using the Pathfinder algorithm, and subjected to keyword K-means clustering analysis with a G-index parameter set to K = 10.

## 3. Results

### 3.1. Trends in Annual Publications

**Figure 2:**
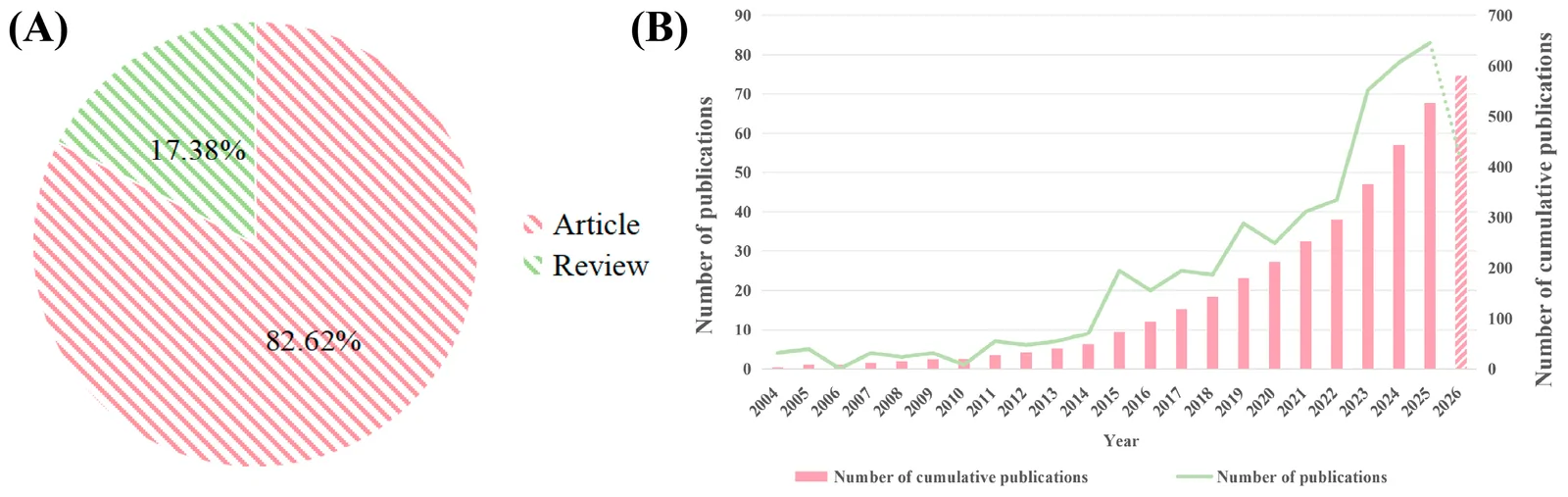
Document types and annual and cumulative publication trends. (**A**) Document type distribution. (**B**) Global annual and cumulative publication output from 2004 to 2025. The partial-year 2026 data (1 January–1 April) are shown as a green dashed line and hatched bars but were excluded from trend analysis, stage classification, and fitting.

A total of 581 publications, retrieved from WoSCC, Scopus, and PubMed, were analyzed, with research articles outnumbering review articles (Figure 2A). As shown in Figure 2B, annual publication output fluctuated but increased overall, while the cumulative output rose steadily. Based on changes in annual output, the complete years from 2004 to 2025 were divided into three phases. Research activity was limited during 2004–2014, increased steadily during 2015–2020, and expanded rapidly during 2021–2025. The minor fluctuation around 2019 did not alter the overall upward trend.

By 1 April 2026, 53 publications had been retrieved for the partial year. Because the 2026 data were incomplete, these records were retained in the final dataset but excluded from annual trend visualization, stage classification, and trend fitting. The partial-year output was consistent with the continued growth observed in the field.

### 3.2. Analysis of Countries/Regions

**Figure 3:**
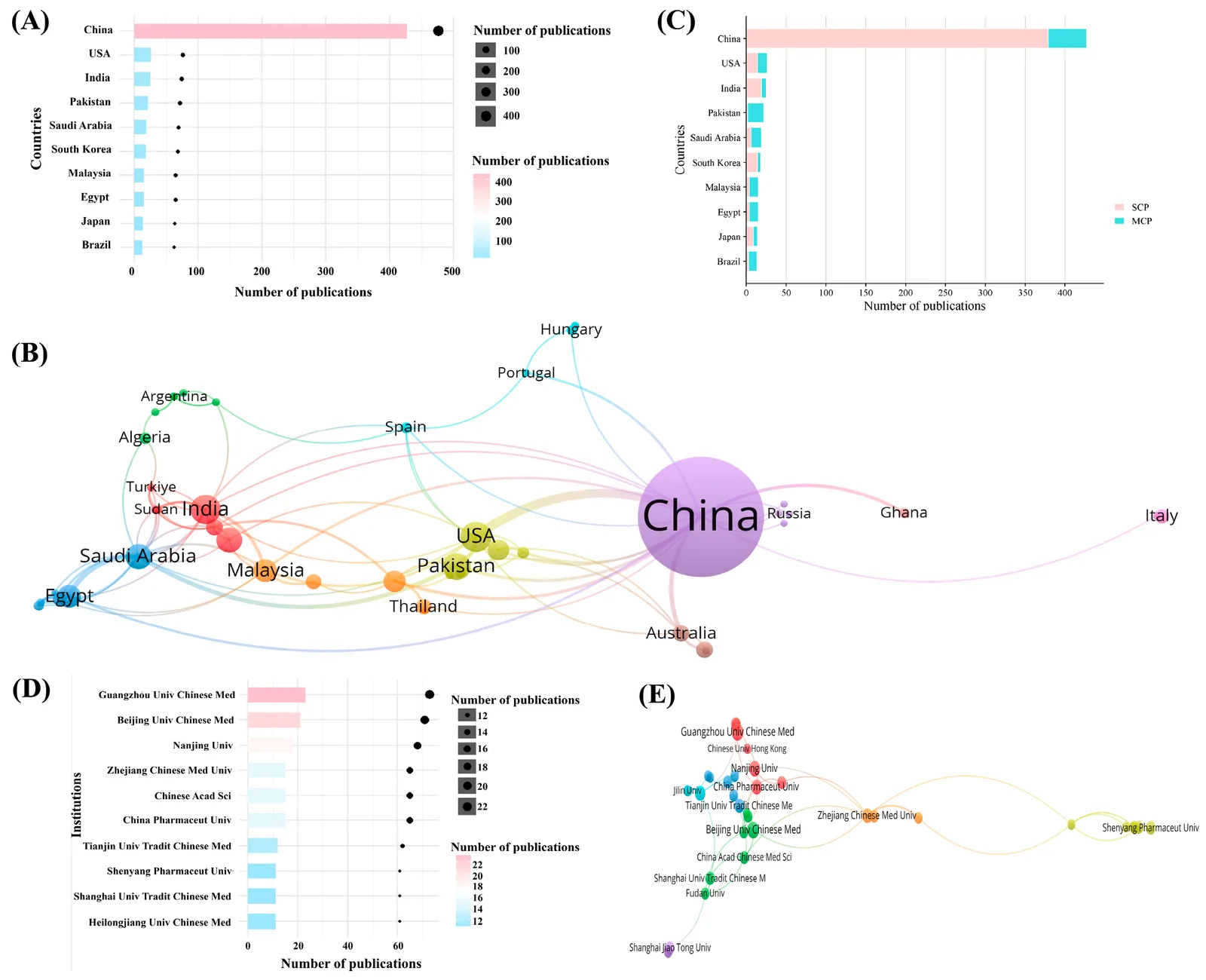
Academic contributions and collaboration networks of countries and institutions. (**A**) Ten most productive countries. (**B**) Country collaboration network. (**C**) SCP and MCP among the ten most productive countries/regions. (**D**) Ten most productive institutions. (**E**) Institutional collaboration network.

Country-level analysis revealed a pronounced geographic concentration of research output. China led in publication output, total citations, and H-index, while East Asia, North America, and South Asia constituted the main contributing regions (Figure 3A; Table S1). Rankings based on AC/P did not fully align with publication output, indicating a difference between research productivity and per-publication influence. In the collaboration network, China occupied a central position, with China–US collaboration representing the strongest international linkage (Figure 3B). Among the ten most productive countries, Chinese publications were predominantly SCP, whereas Pakistan, Malaysia, and Brazil showed relatively higher proportions of MCP, reflecting distinct patterns of domestic research and international collaboration (Figure 3C; Table 1).

### 3.3. Analysis of Contributing Institutions

A total of 870 institutions worldwide contributed to research on NPs-based approaches for HUA. Research output was concentrated in a limited number of institutions, particularly universities of TCM in China. Guangzhou University of Chinese Medicine had the highest publication output (Figure 3D; Table S2). The institutional collaboration network showed that a small group of core institutions occupied central positions, with close links between TCM universities and related research institutes (Figure 3E). Nanjing University had the highest AC/P despite a moderate publication output, indicating that institutional productivity and per-publication influence were not fully aligned.

### 3.4. Author and Co-Cited Author Analysis

**Figure 4:**
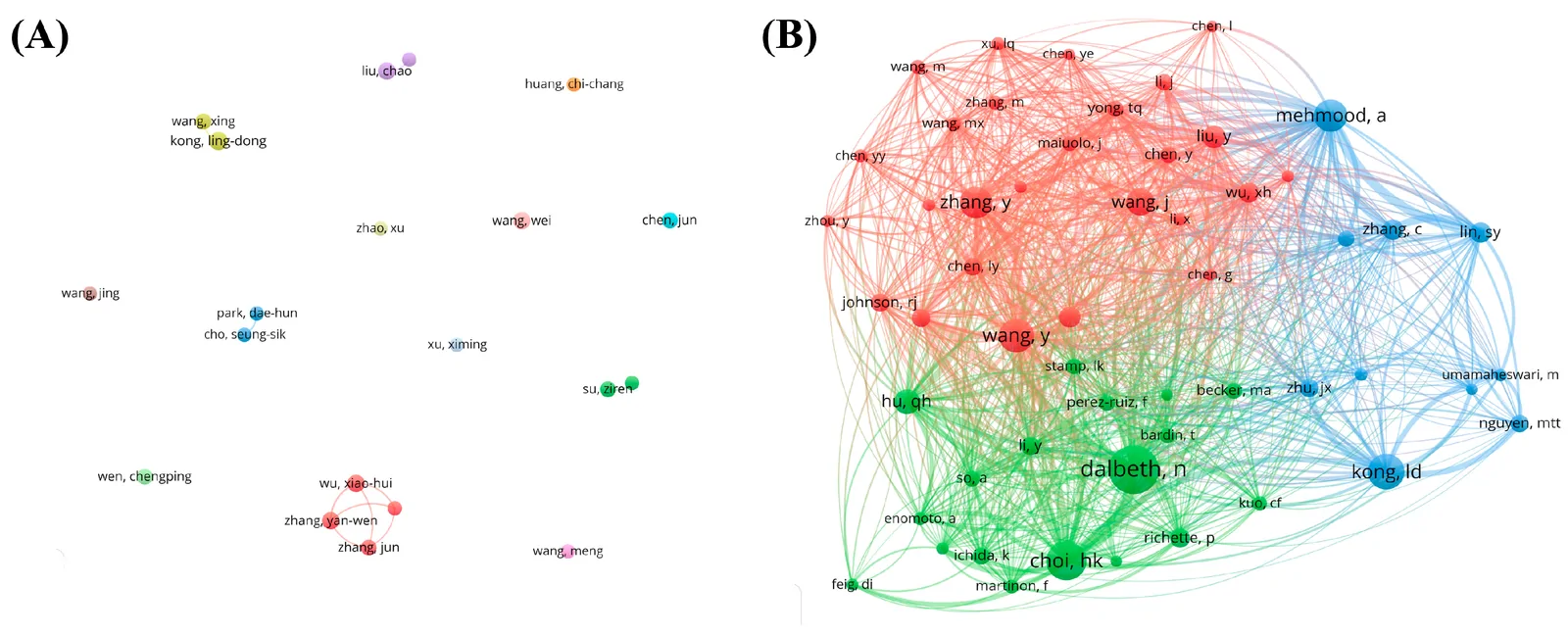
Author and co-cited author collaboration networks. (**A**) Collaboration network among authors. (**B**) Collaboration network among co-cited authors.

Author analysis showed that 3421 authors contributed to research on NPs for HUA, with publication output concentrated within a small group of core authors (Figure 4A; Table S3). Kong Ling-Dong was the most prolific author, whereas each of the ten most productive authors published six to eight articles. This pattern indicated that the field was driven by multiple research teams rather than dominated by a single author. The co-citation network identified Dalbeth N and Choi HK as amongst the most frequently co-cited authors (Figure 4B; Table S3). The limited overlap between prolific and highly co-cited authors indicated that publication productivity and co-citation influence were not fully aligned.

### 3.5. Analysis of Journals and Co-Cited Journals

**Figure 5:**
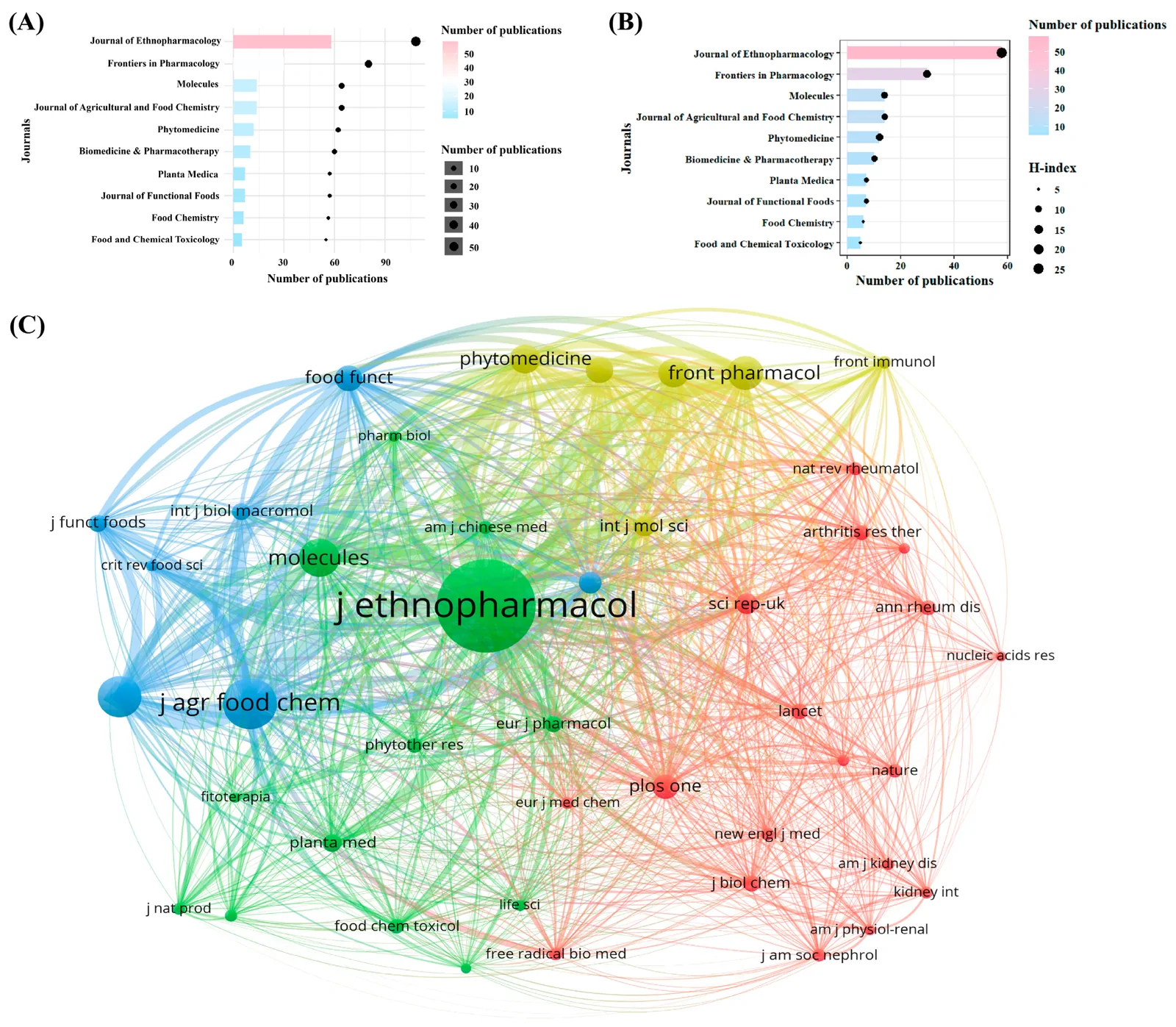
Contributions and collaborations of journals. (**A**) Top 10 most productive journals. (**B**) Top 10 journals ranked by H-index. (**C**) Co-cited journal network.

The 581 publications were distributed across 234 journals, although output was concentrated in a limited number of core sources (Figure 5A; Table S4). The H-index analysis showed a similar concentration of journal-level influence (Figure 5B). In the co-citation network, the *Journal of Ethnopharmacology*, *Journal of Agricultural and Food Chemistry*, and *Frontiers in Pharmacology* occupied central positions based on TLS (Figure 5C). Most frequently co-cited journals were ranked in JCR Q1 or Q2, indicating that research in this field was mainly disseminated through medium- to high-impact journals.

### 3.6. Analysis of Cited Articles and Co-Cited References

Table 2 lists the ten most highly cited publications in the dataset. Five were primary experimental or clinical studies [44,45,46,47,48], and five were reviews [49,50,51,52,53]. These publications reflect the thematic development of the field. Early studies were focused on XOD inhibition and the experimental validation of traditional medicines and medicinal plants [46,48]. Subsequent work expanded to antioxidant activity, structure–activity relationships of natural compounds, urate transport, and multi-target mechanisms [45,47,50,51,52,53]. More recent studies have focused on gut microbiota and clinical translation [44,49]. Collectively, these highly cited publications provided experimental, methodological, mechanistic, and clinical foundations for the field, and their thematic progression was broadly consistent with the keyword trends identified in this study.

The rankings of co-cited references are presented in Table S6. The core co-cited references converged on UA metabolism, XOD regulation, and NPs-based interventions, reflecting a relatively coherent knowledge base in this field [48,54,55,56].

### 3.7. Keyword and Hotspot Analysis

**Figure 6:**
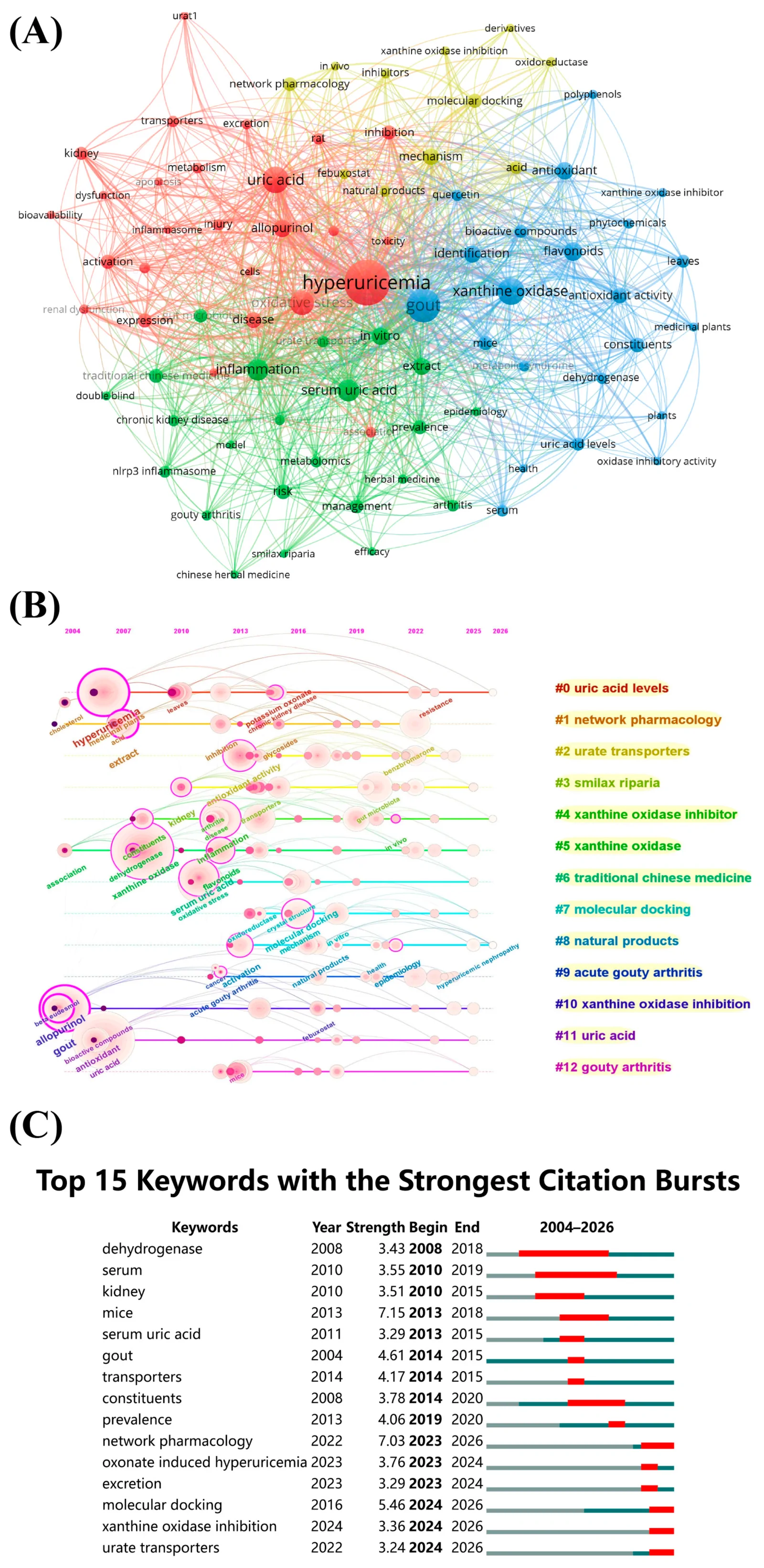
Visualization of co-occurring keywords. (**A**) Keyword co-occurrence network. (**B**) Keyword timeline visualization. (**C**) Top 15 keywords with the strongest citation bursts.

The keyword co-occurrence network comprised 90 nodes. The core keywords included “HUA”, “gout”, “XOD”, and “UA” (Figure 6A). The keyword timeline showed that research expanded from early studies of TCM and XOD to urate transporters, network pharmacology, molecular docking, and gut microbiota (Figure 6B). Burst analysis further indicated a shift from disease- and pathology-oriented research toward technical applications and mechanistic validation. The 15 keywords with the strongest bursts are presented in Figure 6C. “Molecular docking”, “XOD inhibition”, and “urate transporters” remained in the burst phase in 2024, indicating that target identification, mechanistic investigation, and technical validation remained important research directions (Table S7).

## 4. Discussion

### 4.1. Analysis of Literature Types and Annual Output Trends

The distribution of the literature types indicated that research on NPs for the treatment of HUA was predominantly characterized by original empirical studies. The academic community had primarily focused on experimental investigations aimed at elucidating the mechanisms of action of NPs, such as active ingredient isolation [57] and target validation [58], rather than merely summarizing existing knowledge. The annual publication output in this field from 2004 to 2026 exhibited distinct phases of growth. From 2004 to 2014, the volume of publications remained relatively low, largely due to insufficient understanding of the pathological mechanisms of HUA [59,60], the relatively low global incidence of HUA [61], and the limited recognition of the therapeutic potential of NPs. Between 2015 and 2020, publication volume increased compared with the previous phase, a trend closely linked to the growing awareness of the clinical hazards of HUA, including its role in inducing gout [62] and chronic kidney disease [10]. During this period, the unique advantages of NPs, such as multi-target activity and low adverse effects, attracted greater academic attention, thereby contributing to a steady rise in related studies. From 2021 to 2025, the field experienced rapid expansion, driven by heightened global awareness of metabolic diseases, particularly the associations between HUA and obesity [27] and diabetes [63], in the aftermath of the COVID-19 pandemic. The increasing demand for safe and effective long-term treatment options in clinical practice had further stimulated in-depth investigations into the mechanisms of action of NPs. As of 1 April 2026, 53 related papers had been published that year, sustaining the strong growth momentum observed from 2021 to 2025. It is anticipated that this number will continue to rise substantially throughout the year, underscoring the sustained research vitality and considerable development potential of this field.

### 4.2. Country/Region Analysis

China occupied a leading global position in both research output and overall influence in the field of NPs-based therapies for HUA, with its core leadership role clearly evident. Its scholarly contributions extended to neighbouring countries, establishing China as the central driver of NPs research in East Asia. As shown in Table S1, although China ranked first in publication volume, its AC/P remained substantially lower than those of the USA, Japan, and India. This disparity suggested that the academic impact and international visibility of individual studies require further enhancement, potentially due to limitations in research depth and dissemination breadth. These findings indicated that, while sustaining a high publication volume, greater emphasis should be placed on deepening the mechanistic exploration and clinical relevance of research, particularly through mechanistic investigations and the clinical validation of NPs, in order to augment the scholarly influence of individual contributions. The SCP and MCP analyses showed that most Chinese publications were produced through domestic research. Broader high-quality international collaboration may help increase the visibility and citation impact of individual publications. Regarding international collaboration, China’s partnership with the USA was the most robust, positioning both countries as central nodes in regional research networks. Such cooperation provided a critical pathway to narrow the per-publication impact gap between China and other leading countries, while simultaneously accelerating the global translation and adoption of NPs for HUA treatment.

### 4.3. Institutional Analysis

Globally, numerous institutions had contributed to research on the application of NPs for the treatment of HUA. Among the top ten institutions by research output, universities specializing in TCM accounted for an impressive 80%, underscoring their dominant leadership in this field. This leadership stems from their abundant NPs resources and the extensive, long-standing research foundation in pharmacology. The research conducted by these institutions consistently centred on TCM formulas, plant extracts, and other NPs, aligning closely with the clinical demand for safe, multi-target therapeutic approaches to HUA. This emphasis provides a solid basis for an in-depth exploration of the mechanisms of action and facilitates the translational application of NPs research into clinical practice. Furthermore, the development of institutional collaboration networks had enabled the integration of the clinical expertise of TCM universities with advanced molecular biology techniques offered by partner institutions, such as liquid chromatograph mass spectrometry [64] and multi omics analysis [65]. This synergy between resources and technologies had substantially expanded and deepened the research scope, while strengthening the theoretical underpinnings of HUA therapy. In terms of academic impact, Nanjing University ranked highest in AC/P, markedly surpassing other institutions. Its research exhibited a stronger focus on mechanistic depth and clinical relevance, exemplified by comprehensive analyses of the inhibitory mechanisms of TCM formulas on XOD [66,67]. These qualities enhanced the scholarly value of its findings, bolster their potential for recognition by high-impact international journals, and highlight the institution’s comparative advantage in research quality.

### 4.4. Authors and Co-Cited Authors

Researchers worldwide had actively engaged in studies on the use of NPs to treat HUA, highlighting the field’s high degree of openness and the broad participation of diverse stakeholders. The distribution of highly productive authors appeared relatively even, with the cumulative number of publications by the top ten authors ranging from six to eight papers. No single author demonstrated absolute dominance in research output; instead, progress in the field was driven primarily by multiple small, collaborative teams and independent investigators. Within this cohort, Kong Ling-Dong led in publication volume, while Liu Chao, Wang Wei, and several others formed a persistently active core research group. Co-citation network analysis further revealed key authoritative scholars whose work underpins the field’s knowledge base. Notably, Dalbeth N and Choi HK had produced pivotal studies elucidating the pathophysiological mechanisms of HUA and informing its clinical management [68,69]. Kong LD, affiliated with Nanjing University, was dedicated to integrating TCM theory with modern pharmacological methods to investigate, at a mechanistic level, the UA-lowering effects of TCM formulas such as Ermiao Wan. This line of inquiry constituted a central focus of NPs-based therapy research [48] and had positioned him among the top three most frequently co-cited authors. Collectively, the contributions of these influential scholars had established a solid, interdisciplinary theoretical framework for advancing the prevention and treatment of HUA, particularly through TCM-derived NPs.

### 4.5. Journals and Co-Cited Journals

An analysis of the distribution of journals and co-cited journals in the field of NPs-based therapy for HUA indicated that research published between 2004 and 2026 was concentrated in a relatively small number of medium-to-high-impact journals. The top ten journals by publication volume accounted for 28.06% of all articles, suggesting the establishment of relatively mature core channels for academic dissemination. Among these, the *Journal of Ethnopharmacology*, with its focus on ethnopharmacology and the therapeutic application of NPs, aligned closely with the theme of “NPs for the treatment of HUA”, making it the most influential platform for knowledge dissemination in this domain. This was followed by *Frontiers in Pharmacology* and *Phytomedicine*, which emphasized pharmacological research frontiers and phytomedicine studies, respectively. They highlighted the central role of “pharmacological mechanism analysis of NPs” in advancing the field. Notably, the presence of journals such as the *Journal of Agricultural and Food Chemistry* and *Food Chemistry*, both dedicated to food science and agricultural chemistry, emphasized that “chemical composition analysis of NPs” constituted a crucial foundation for research in this area. It also reflected increasing scholarly interest in NPs derived from food sources, such as curcumin [70] and resveratrol [71], given their strong relevance to dietary interventions. A co-citation journal network analysis further delineated the field’s core knowledge structure, revealing that the cited literature is primarily clustered into four thematic areas, ethnopharmacology, chemical composition analysis, pharmacological mechanism investigation, and clinical application research.

### 4.6. Cited Articles and Co-Cited References

The highly cited literature indicates that research on NPs for HUA has progressed from enzyme-centred screening toward broader mechanistic and translational investigation. Kong et al. [48] and Umamaheswari et al. [46] represent early studies focused on XDH/XOD inhibition by TCM and medicinal plants. Palsamy and Subramanian [45] and Zhao et al. [47] extended this work to antioxidant activity, metabolic regulation, and differences among NPs constituents. However, Palsamy and Subramanian [45] used a diabetic animal model; their findings should therefore not be interpreted directly as evidence of anti-HUA activity.

Kostić et al. [51] and Mehmood et al. [53] provided methodological and chemical frameworks through their reviews of XOD activity assessment, natural inhibitor classes, and structure–activity relationships. Sahebkar [50] and Tao et al. [52] further broadened the field from single-target inhibition to metabolic regulation, urate transport, and renal protection. Wang et al. [49] summarized the role of gut microbiota in UA metabolism and excretion, highlighting intestinal regulation as an emerging research direction. The randomized controlled trial by Panahi et al. [44] provided clinical evidence relevant to the UA-lowering effects of curcumin. However, the participants had non-alcoholic fatty liver disease rather than specifically diagnosed HUA. Overall, these highly cited publications and their co-citation relationships reflect the development of the field from XDH/XOD-focused screening and preclinical validation toward antioxidant and multi-target mechanisms, gut–kidney axis regulation, and clinical translation. Nevertheless, citation prominence should not be equated with clinical efficacy, and further clinical studies in patients with HUA are needed.

### 4.7. Keywords and Trends

This study conducted a keyword analysis of the literature on NPs interventions for HUA published between 2004 and 2026, identifying both core research themes and their evolutionary trajectories. Co-occurrence network analysis revealed that “HUA”, “gout”, “XOD”, and “UA” form the conceptual foundation of the field, delineating the mechanistic pathway of “XOD inhibition → UA reduction → HUA improvement”. The high centrality of “oxidative stress” and “inflammation” highlighted their dual roles as fundamental pathological mechanisms and therapeutic targets for NPs-based interventions. Temporal trend analysis indicated a phased evolution; the early stage (2004–2010) concentrated on mechanisms of traditional drugs, the mid-stage (2011–2015) shifted toward NPs and oxidative stress, and the most recent stage (2016–2026) had seen the emergence of “gut microbiota” and “molecular docking”, reflecting a transition toward microbiome focused and systems biology approaches [72]. Burst detection analysis further demonstrated that “network pharmacology” (burst intensity: 7.03) and “molecular docking” [73,74] (burst period, 2023–2026) had become contemporary driving forces, enabling more precise identification and characterization of NPs targets. Looking ahead, with the continuous advancement of multi omics strategies and molecular docking techniques, mechanistic research on bioactive components from TCM will be conducted with greater depth and accuracy. Meanwhile, in-depth studies on novel therapeutic targets, including gut microbiota regulation and inflammatory signalling pathways, have offered promising new insights for the clinical intervention of related diseases.

### 4.8. NPs and HUA

#### 4.8.1. Flavonoids

Building upon a review of studies conducted prior to 2019 [75], substantial progress had been made between 2020 and 2026 in the investigation of flavonoids for HUA treatment, as summarized in Table S8. Recent advances have encompassed the screening and mechanistic validation of potent inhibitors, enhancement of bioavailability, identification of novel targets such as UA transporters, and elucidation of multi-target synergistic mechanisms. Zhaoyu, Wu et al. employed an online capillary electrophoresis-immobilized enzyme microreactor (IMER) integrated with molecular docking to assess the inhibitory activity of ten flavonoids against XOD, reporting that dihydroquercetin, quercetin, biochanin A, and epicatechin exhibited strong inhibitory effects, with molecular docking binding energies closely matching IMER assay outcomes [76]. Aguirre Einy Nallybe Bedoya et al. investigated three chromene flavanones (CFs) isolated from *Dalea boliviana*, and in vitro activity profiling combined with molecular docking confirmed that (2S)-5,2′-dihydroxy-6″,6″-dimethylchromeno-(7,8:2″,3″)-flavanone and (2S)-5,2′-dihydroxy-6″,6″-dimethylchromeno-(7,8:2″,3″)-3′-prenylflavanone demonstrated potent XOD inhibition, whereas obovatin exhibited weaker activity, suggesting that specific CF structural types may serve as promising scaffolds for developing novel XOD inhibitors [77]. Liu Yang et al. isolated and characterized three flavonoids from *Perilla frutescens* leaves, namely (2S)-5,7-dimethoxy-8,4′-dihydroxyflavanone, 2′,4′-dimethoxy-4,5′,6′-trihydroxychalcone, and (Z)-4,6-dimethoxy-7,4′-dihydroxyaurone, among which the second compound exhibited the strongest XOD inhibitory activity [57]. Linani Abderahmane et al. systematically evaluated flavonoid (Cae) and alkaloid (CaK) extracts from *Cupressus sempervirens* L. along with six derived compounds, finding that CaK showed significantly higher XOD inhibitory activity than Cae, while computational analysis identified lignan and amentoflavone as potent inhibitors, cupressuflavone as moderately active, and isohyperforin, hinokiflavone, and neolignan as less active [78]. Addressing the common limitation of poor bioavailability, Song Danni et al. applied solid dispersion (SD) technology to kaempferol (KPF), markedly improving its solubility and in vivo bioavailability, which enhanced its ability to reduce SUA levels and mitigate renal injury [79]. Yang Bendong et al. further demonstrated that naringenin exerts anti-hyperuricemic effects by modulating renal UA transporter expression to promote UA excretion, while simultaneously inhibiting the Toll-like receptor 4 (TLR4)/Nuclear factor κB (NF-κB) pathway to reduce renal inflammation, supporting its potential as a functional food ingredient or dietary supplement [58]. Toyoda Yu et al. examined 13 dietary flavonoids and related compounds for their URAT1 inhibitory activity, identifying fisetin and quercetagetin as the most potent inhibitors, thereby providing new perspectives for the development of agents targeting UA reabsorption. Multiple studies have highlighted the capacity of flavonoids to alleviate HUA-associated renal injury via multi-target synergistic mechanisms [80]. ALRashdi, Barakat M. et al. isolated six flavonoid compounds from *Monolluma quadrangula*, including kaempferol-7-O-β-D-glucopyranoside, kaempferol-3-O-β-D-glucopyranosyl (6 → 1)-α-L-rhamnopyranoside, luteolin-3′,4′-di-O-β-D-glucopyranoside, luteolin-7-O-β-D-glucopyranoside, chrysoeriol, and luteolin, which act synergistically through three mechanisms: inhibiting XOD to reduce UA production, modulating renal UA transporters, and alleviating oxidative stress by regulating MDA, GSH and SOD levels while suppressing inflammatory responses [81]. Lv Shuai et al. revealed that anthocyanins lower UA levels by inhibiting XOD and adenosine deaminase to suppress UA synthesis, restoring gut microbiota structure to enhance UA metabolism, and exerting anti-inflammatory activity [82]. Additionally, SuJung, Hsu et al. developed an intestinal liver microenvironment biomimetic model combined with targeted metabolomics to rapidly evaluate the bioavailability and UA-lowering efficacy of apigenin, which significantly reduced UA levels in the basolateral compartment and demonstrated potential as a preventive nutritional supplement [83].

#### 4.8.2. Phenolic Acids

Building upon previous reports summarizing advances in phenolic acid compounds up to 2022 [84], this section reviews notable progress achieved from 2023 to 2026 in elucidating the mechanisms of action and therapeutic potential of phenolic acids for HUA management. Lou Ye et al. demonstrated that ferulic acid mitigates HUA-associated pathological alterations by selectively inhibiting XOD activity, thereby reducing UA biosynthesis [85]. Li Kui et al. employed bioactivity-guided separation to isolate phenolic acids with potent XOD inhibitory activity from the fruits of *Chaenomeles speciosa* (Sweet) Nakai, identifying chlorogenic acid, caffeic acid, p-coumaric acid, and protocatechuic acid as the principal active constituents, whose UA-lowering effects were mediated predominantly via XOD inhibition [86]. GuoYi, Tang and colleagues utilized ultra performance liquid chromatography coupled with diode array detection (UPLC-DAD) to establish a chemical fingerprint profile of a renal herbal formula (RHF) and characterize its major components. Their findings indicated that RHF alleviates HUA through a dual mechanism, markedly decreasing SUA concentrations while concurrently modulating the p53-mediated apoptosis pathway and inhibiting the NF-κB inflammatory pathway. Chlorogenic acid, neochlorogenic acid, cryptochlorogenic acid, and isochlorogenic acids A–C were identified as the characteristic bioactive molecules, suggesting that RHF and its chlorogenic acid analogues are promising candidates for novel HUA interventions [87]. Liang Shaoyu et al. investigated Phyllanthi Fructus as a model herb, integrating network pharmacology, molecular docking, and molecular dynamics simulations to comprehensively elucidate its multi pathway anti-HUA mechanisms. Results demonstrated that Phyllanthi Fructus inhibits XOD activity to suppress UA production, regulates UA transporter expression to facilitate UA excretion, and attenuates oxidative stress alongside inflammatory responses. Active component analysis confirmed that phenolic acids (ellagic acid, gallic acid) and flavonoids (quercetin, epigallocatechin gallate) constitute the pharmacological basis of Phyllanthi Fructus efficacy, providing robust evidence for its development as a natural-product-based anti-HUA formulation [88]. Furthermore, Tipsuwan Wachiraporn et al. reported that rosmarinic-acid-rich extracts derived from perilla seed meal possess strong antioxidant properties and XOD inhibitory capacity, underscoring their potential for advancement into clinical translational research for HUA treatment [89].

#### 4.8.3. Alkaloids

Previous reviews have summarized the therapeutic applications of alkaloid compounds for HUA prior to 2021 [90]. This section outlines advances from 2022 to 2026 in the identification and characterization of alkaloids exhibiting anti-HUA activity. Chen, Qinghong et al. screened through computational biomedical modelling and found that chelerythrine in the Miao medicine ‘Simao pills’ may reduce UA synthesis by inhibiting xanthine dehydrogenase (XDH). They also predicted that endothelial nitric oxide synthase (NOS3) is a potential target for treating HUA. These results need to be further verified by experiments [91]. Ai Gaoxiang’s group demonstrated that palmatine, derived from Cortex Phellodendri Amurensis, significantly ameliorates potassium oxonate/hypoxanthine-induced HUA in animal models by markedly suppressing renal inflammatory mediators and oxidative stress markers such as XOD and adenosine deaminase (ADA), while simultaneously regulating UA transporters, thereby achieving dual effects of UA reduction and renal protection and expanding its potential clinical applications [92].Yukai, Zhang et al. combined in vitro and in vivo assays with molecular docking to elucidate the anti-HUA activity of Nuciferine. In vitro experiments revealed potent URAT1 inhibition, whereas in vivo studies showed significant decreases in SUA levels and amelioration of kidney injury in HUA mice through the modulation of URAT1 and Glucose transporter 9 (GLUT9) expression, coupled with inhibition of the TLR4/NF-κB inflammatory pathway. Molecular docking further demonstrated stable binding interactions with the URAT1 protein, supporting their promise as candidates for anti-HUA therapy and renal function preservation [93]. LiLu’s team confirmed, via both in vitro and in vivo studies, that piperine regulates URAT1 and GLUT9 by modulating the protein kinase B/mammalian target of rapamycin (AKT/mTOR) signalling pathway, thereby effectively preventing the pathological progression of HUA and demonstrating considerable translational potential [94]. Xu Lieqiang et al. integrated network pharmacology with experimental validation to characterize the multi-target mechanisms of Cortex Phellodendri in HUA treatment. Network analysis identified berberine as the principal active alkaloid, and experimental results confirmed that Cortex Phellodendri inhibits XOD activity to lower UA via a “multi-component, multi-target, multi-pathway” synergistic mechanism, while simultaneously reversing renal injury through anti-inflammatory and anti-apoptotic actions [95].

#### 4.8.4. Quinones

Shenwei, Hou et al. demonstrated, using a rat model, that emodin, an active component of TCM, effectively lowers SUA levels by promoting UA excretion, thereby ameliorating HUA and alleviating gout symptoms [96]. Zhaoqing, Meng’s team established a mouse model of HUA with concomitant kidney injury induced by adenine and ethambutol and systematically evaluated the therapeutic effects of rhein ester, which significantly decreased SUA via inhibition of XOD activity and reduced the production of pro-inflammatory cytokines, thereby mitigating renal injury and highlighting its translational potential as an anti-HUA and renoprotective agent [97]. Cloete Stephanus J. et al. screened quinone derivatives for XOD inhibitory activity in vitro and identified that naphthoquinones (e.g., juglone, menadione) and tricyclic derivatives (9,10-phenanthrenequinone, quinalizarin) displayed substantial inhibitory effects, providing a molecular basis for the rational design of novel quinone-based XOD inhibitors [98]. Gao Tianshu’s group, employing a potassium oxonate-induced HUA mouse model, elucidated the multi-target mechanisms of N-(9,10-anthraquinone-2-ylcarbonyl) derivatives, which reduce UA production through XOD inhibition, co-regulate UA transporters to facilitate UA excretion, and suppress key mediators of the NLR family pyrin domain containing 3 (NLRP3) inflammasome pathway to attenuate renal injury, collectively suggesting promising avenues for anti-HUA drug development [99].

#### 4.8.5. Terpenoids

Hlophe Nomadlozi Blessings et al. employed an adenine–gentamicin-induced HUA rat model with concomitant renal toxicity to demonstrate that lanosterol triterpenes markedly improve renal function parameters and UA metabolism indicators while alleviating histopathological kidney damage. These findings highlight the dual therapeutic potential of lanosterol triterpenes in anti-HUA therapy and renal protection, offering promising prospects for the prevention and treatment of kidney diseases [100]. Chen Yan et al. reported that total saponins from *Dioscorea septemloba* Thunb selectively upregulate the expression of organic anion transporting polypeptide 1A1 (OATP1A1), thereby activating the intestinal hepatic UA excretion pathway and effectively reducing SUA levels in HUA rats. This non-renal clearance mechanism circumvents the renal toxicity risks associated with conventional uricosuric agents, providing a novel strategy for HUA management [101]. Zhou Qi’s group demonstrated that total saponins from *Dioscorea nipponica* Makino bidirectionally modulate UA transporter expression, thereby promoting UA excretion, lowering SUA concentrations, and supporting their potential both as TCM for HUA and as candidate agents for gouty arthritis [102]. Hou Shixiang et al. revealed that iridoid glycosides from *Paederia scandens* (Lour.) protect renal function in a yeast potassium oxonate-induced UA nephropathy model by lowering SUA, suppressing inflammatory responses, and modulating immune function. These effects are mediated by coordinated upregulation of nitric oxide synthase-1 (NOS-1) and downregulation of tumour necrosis factor-α (TNF-α) and transforming growth factor-β1 (TGF-β1), thereby slowing the progression of HUA-related kidney injury [103]. Lin Xuan’s team confirmed that quinoa (*Chenopodium quinoa* Willd.) bran saponins alleviate HUA by simultaneously regulating the phosphoinositide 3-kinase/protein kinase B/NF-κB signalling pathway to inhibit inflammation and modulating UA transporter expression to enhance UA metabolism, thereby concurrently reducing kidney injury [104]. Hu Binyuan et al. validated the therapeutic potential of triterpenoid compounds through both in vitro and in vivo experiments. The in vitro assays demonstrated enhanced UA excretion, while the in vivo studies confirmed reduced SUA levels, providing additional evidence for their role in HUA treatment [105]. Wu Xiaohui’s group investigated pallidifloside D from *Smilax riparia*, confirming its anti-HUA activity via selective regulation of UA reabsorption transporters [106].

#### 4.8.6. Polysaccharides

Zhang Nanxin et al. employed a HUA mice model induced by both potassium oxonate and allopurinol to demonstrate that Polygonati rhizoma polysaccharides exert both anti-HUA and renoprotective effects by significantly lowering SUA levels and improving renal function parameters [107]. Sun Lirui’s team investigated the underlying mechanism of rockweed (*Saccharina japonica*) polysaccharides and revealed that they effectively attenuate HUA-induced renal fibrosis by specifically inhibiting activation of the Janus kinase 2/signal transducer and activator of transcription 3 (JAK2/STAT3) signalling pathway [108]. These findings provide compelling experimental evidence supporting the therapeutic potential of natural polysaccharides in HUA management.

**Figure 7:**
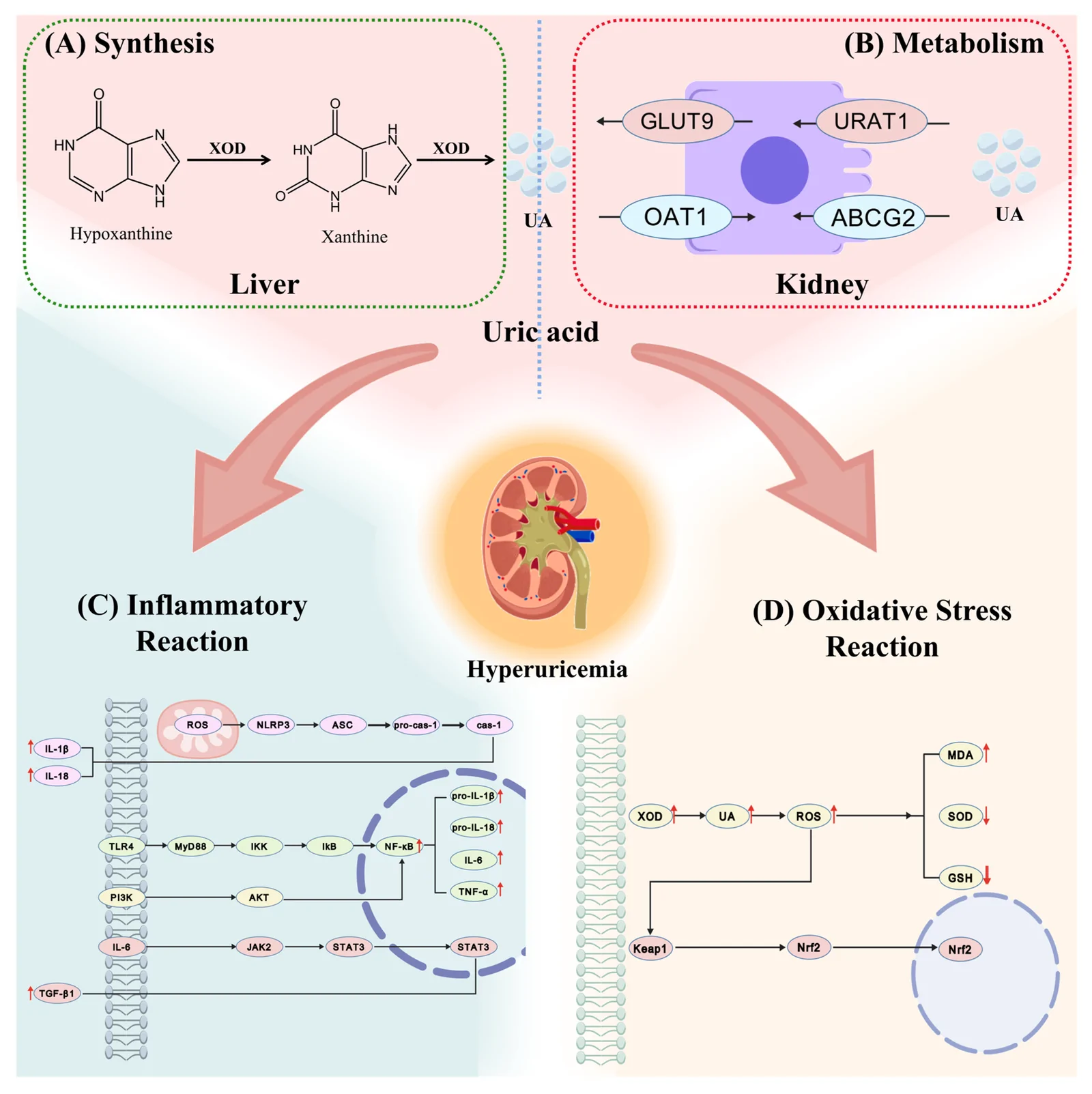
Mechanism diagram of natural compounds for HUA treatment. (**A**) UA synthesis. (**B**) UA metabolism. (**C**) Inflammatory reaction. (**D**) Oxidative stress.

Based on the above literature, the mechanisms of action of the six classes of natural compounds for HUA treatment are summarized in Figure 7.

### 4.9. Clinical and Translational Implications

The bibliometric findings do not establish the therapeutic efficacy of any NPs, but they may help define priorities for future research. XOD inhibition, URAT1/GLUT9 regulation, gut microbiota, oxidative stress, and inflammatory pathways were recurrent themes in the literature. These findings may guide the screening of NPs and candidate compounds, target validation, structure–activity analysis, and selectivity assessment. Candidates supported by reproducible experimental evidence should be further evaluated for pharmacokinetics, bioavailability, dose–response relationships, toxicity, and renal safety to facilitate translation from basic research.

Clinical evidence in this field remains limited. Future clinical trials should therefore enrol patients with well-defined HUA and prespecify outcomes including SUA, renal function, and safety. Concomitant medication, dose, and follow-up duration should also be considered. The development of evidence-based phytomedicines requires botanical authentication, species identification, marker-compound characterization, batch consistency, defined dosage and formulation, and appropriate quality-control standards. Integrating bibliometric priorities with pharmacological, pharmacokinetic, toxicological, and clinical evidence may improve the reproducibility and translational value of candidate NPs. Thus, bibliometric findings should be regarded as a guide to research priorities rather than a substitute for evidence of efficacy and safety.

## 5. Limitations and Outlook

Although bibliometric analysis provides a quantitative perspective on NPs-based therapy for HUA, several limitations should be acknowledged. The study covered publications from 1 January 2004 to 1 April 2026. Because only partial-year data were available for 2026, these records were excluded from annual trend analysis and model fitting. Recent publications also had limited time to accumulate citations, and studies published after the search date were not captured.

Only English-language articles and reviews indexed in Web of Science, Scopus, and PubMed were included. Books, book chapters, conference materials, and other grey literature were excluded or not retrieved. Differences in database coverage, indexing practices, metadata completeness, and self-citation may have affected the bibliometric indicators. Citation-based measures may also be influenced by publication age and the rapid development of the field. Therefore, highly cited publications and emerging topics should be interpreted as indicators of scholarly attention rather than direct evidence of clinical efficacy. Future studies should incorporate broader databases, regularly updated datasets, and pharmacological and clinical evidence.

Currently, research into the pathological mechanisms of HUA, including inflammation, oxidative stress, gut microbiota, and UA transporter regulation, is advancing rapidly. NPs, particularly plant-derived compounds such as flavonoids, have shown considerable potential owing to their multi-target modes of action. Future research in this field is likely to focus on elucidating the molecular targets and signalling pathway mechanisms of bioactive constituents; integrating systems biology approaches (e.g., multi omics analysis and organoid or biomimetic models) to strengthen mechanistic understanding and safety evaluation; enhancing structural optimization; and developing innovative delivery systems (e.g., nanotechnology and solid dispersions) to overcome bioavailability limitations. In parallel, improving bibliometric methods will further facilitate innovation and drive breakthroughs in NPs-based therapies for HUA.

## 6. Conclusions

This study performed a bibliometric analysis of 581 publications on the treatment of HUA with NPs, employing Bibliometrix, VOSviewer, and CiteSpace. It systematically delineated the evolutionary trajectory and future prospects of this research field from 2004 to 2026. The results indicated a sustained exponential increase in related publications, with cumulative growth closely fitting mathematical models, thereby confirming the growing emphasis on NPs-based interventions to address the global burden of HUA. China has become a dominant force in the field with its overwhelming academic contributions, and its extensive international cooperation network highlights the strategic value of integrating local TCM resources with international scientific research paradigms. Through keyword salience analysis, clustering timelines, and co-citation network mapping, this study identified three cutting edge research directions, namely deepening mechanistic studies driven by multi omics technologies, exploring innovative regulatory pathways of the gut–kidney axis, and achieving breakthroughs in the translation of evidence-based medicine. These findings offer important guidance for shaping future research agendas. It is essential to integrate interdisciplinary approaches (e.g., combining metabolomics with metagenomics) and implement rigorous clinical trial designs to accelerate the translation of NPs from mechanistic insights to evidence-based therapies, ultimately providing safer and more cost-effective global solutions for the prevention and treatment of HUA.

## Figures and Tables

**Table 1:** Top 10 countries/regions by SCP and MCP for NPs treatment of HUA from 2004 to 2026.

Rank	Country	Publications	SCP	MCP
1	China	427	379	48
2	USA	26	14	12
3	India	25	19	6
4	Pakistan	22	2	20
5	Saudi Arabia	19	6	13
6	South Korea	18	14	4
7	Malaysia	15	4	11
8	Egypt	15	4	11
9	Japan	14	9	5
10	Brazil	13	3	10

**Table 2:** Top 10 highly cited articles on NPs treatments for HUA from 2004 to 2026.

Rank	Title	Journal	Citations	Publication Year	First Author	JCR	IF (2026)
1	Curcumin Lowers Serum Lipids and Uric Acid in Subjects with Nonalcoholic Fatty Liver Disease: A Randomized Controlled Trial	*Journal of Cardiovascular Pharmacology*	207	2016	Panahi, Yunes	Q2	2.2
2	The gut microbiota as a target to control hyperuricemia pathogenesis: Potential mechanisms and therapeutic strategies	*Critical Reviews in Food Science and Nutrition*	202	2022	Wang, Jing	Q1	8.8
3	Resveratrol, a natural phytoalexin, normalizes hyperglycemia in streptozotocin-nicotinamide induced experimental diabetic rats	*Biomedicine & Pharmacotherapy*	175	2008	Palsamy, P.	Q1	7.5
4	Why it is necessary to translate curcumin into clinical practice for the prevention and treatment of metabolic syndrome?	*Biofactors*	139	2013	Sahebkar, Amirhossein	Q1	5
5	Xanthine Oxidase: Isolation, Assays of Activity, and Inhibition	*Journal of Chemistry*	128	2015	Kostic, Danijela A.	Q2	2.6
6	Xanthine oxidase inhibitory activity of some Indian medical plants	*Journal of Ethnopharmacology*	128	2007	Umamaheswari, Muthuswamy	Q1	5.4
7	Dioscin: A diverse acting natural compound with therapeutic potential in metabolic diseases, cancer, inflammation and infections	*Pharmacological Research*	122	2018	Tao, Xufeng	Q1	10.5
8	Natural compounds with xanthine oxidase inhibitory activity: A review	*Chemical Biology & Drug Design*	109	2019	Mehmood, Arshad	Q2	3.3
9	In Vitro and In Vivo Studies on Adlay-Derived Seed Extracts: Phenolic Profiles, Antioxidant Activities, Serum Uric Acid Suppression, and Xanthine Oxidase Inhibitory Effects	*Journal of Agricultural and Food Chemistry*	95	2014	Zhao, Mouming	Q1	6.2
10	A Chinese herbal medicine Ermiao wan reduces serum uric acid level and inhibits liver xanthine dehydrogenase and xanthine oxidase in mice	*Journal of Ethnopharmacology*	88	2004	Kong, LD	Q1	5.4

## Data Availability

No new data were created or analyzed in this study. Data sharing is not applicable to this article.

## Supplementary Materials

The following supporting information can be downloaded at: https://www.mdpi.com/article/10.3390/ph19081286/s1. Table S1: Top 10 countries/regions for NPs treatment of HUA from 2004 to 2026; Table S2: Top 10 collaborating institutions for NPs treatment of HUA from 2004 to 2026; Table S3: Top 10 most productive authors and co-cited authors in NPs treatment of HUA from 2004 to 2026; Table S4: Top 10 journals on NPs treatment of HUA from 2004 to 2026; Table S5: Top 10 co-cited journals on NPs treatment of HUA from 2004 to 2026; Table S6: Top 10 most co-cited articles on NPs for HUA treatment 2004 to 2026; Table S7: Top 10 high-frequency keywords for NPs treatments for HUA from 2004 to 2026; Table S8: Natural compounds for treating HUA; File S1: Supplementary Methods: Database-Specific Search Strings.

## Abbreviations

ABCG2, ATP-binding cassette transporter G2; AC/P, average citations per paper; GLUT9, Glucose transporter 9; HUA, hyperuricemia; MCP, multiple-country publications; NF-κB, nuclear factor κB; NPs, natural products; SCP, single-country publications; SUA, serum uric acid; TCM, traditional Chinese medicine; TLR4, Toll-like receptor 4; TLS, total link strength; UA, uric acid; URAT1, urate transporter 1; WoSCC, Web of Science Core Collection; XOD, xanthine oxidase.
