# Supplementary material for: Global Trends and Research Hotspots in Natural Products for Hyperuricemia: A Bibliometric Analysis (2004–2026)
Source: Pharmaceuticals (Basel). 2026 Aug 14;19(8):1286. doi: 10.3390/ph19081286 (PMC13517059; doi:10.3390/ph19081286)
Supplement: Supplementary file 1 [file pharmaceuticals-19-01286-s001.zip › pharmaceuticals-4459827-supplementary.pdf]

Table S1 Top 10 countries/regions for NPs treatment of HUA from 2004 to 2026

Rank	Country	Publications	Citations	AC/P	H-index (%)	Total link strength
1	China	427	8139	19.06	46	37
2	USA	26	824	31.69	16	28
3	India	25	595	23.80	10	12
4	Pakistan	22	240	10.91	8	15
5	Saudi Arabia	19	95	5.00	5	22
6	South Korea	18	270	15.00	11	6
7	Malaysia	15	260	17.33	9	14
8	Egypt	15	224	14.93	8	12
9	Japan	14	410	29.29	10	9
10	Brazil	13	248	19.08	6	5

Table S2 Top 10 collaborating institutions for NPs treatment of HUA from 2004 to 2026

Rank	Institutions	Publications	Citations	AC/P	Total link strength
1	Guangzhou Univ Chinese Med	23	363	15.78	7
2	Beijing Univ Chinese Med	21	503	23.95	7
3	Nanjing Univ	18	817	45.39	5
4	China Pharmaceut Univ	15	363	24.20	7
5	Zhejiang Chinese Med Univ	15	318	21.20	11
6	Chinese Acad Sci	15	239	15.93	11
7	Tianjin Univ Tradit Chinese Med	12	188	15.67	3
8	Shanghai Univ Tradit Chinese Med	11	172	15.64	5
9	Shenyang Pharmaceut Univ	11	172	15.64	1
10	Heilongjiang Univ Chinese Med	11	149	13.55	2

Table S3 Top 10 most productive authors and co-cited authors in NPs treatment of HUA from 2004 to 2026

Rank	Authors	Publication	Rank	Co-cited authors	Citation
1	kong, ling-dong	8	1	dalbeth, n	126
2	liu, chao	7	2	choi, hk	101
3	wang, wei	7	3	kong, ld	93
4	wu, xiao-hui	7	4	wang, y	89
5	zhang, yan-wen	7	5	mehmood, a	84
6	chen, jun	6	6	zhang, y	83
7	su, ziren	6	7	wang, j	71
8	wang, xing	6	8	hu, qh	65
9	wen, chengping	6	9	liu, y	60
10	zhang, jun	6	10	wang, x	55

Table S4 Top 10 journals on NPs treatment of HUA from 2004 to 2026

Rank	Journal	publications	citations	AC/P	IF (2026)	H-index (%)	JCR division	Country
1	Journal of Ethnopharmacology	58	1777	30.64	5.4	25	Q1	Ireland
2	Frontiers in Pharmacology	30	557	18.57	4.8	14	Q1	Switzerland
3	Journal of Agricultural and Food Chemistry	14	372	26.57	6.2	10	Q1	United States
4	Molecules	14	310	22.14	4.6	9	Q2	Switzerland
5	Phytomedicine	12	402	33.50	8.3	11	Q1	Germany
6	Biomedicine & Pharmacotherapy	10	476	47.60	7.5	9	Q1	France
7	Planta Medica	7	219	31.29	2.0	6	Q2	Germany
8	Journal of Functional Foods	7	143	20.43	4.0	6	Q2	Netherlands
9	Food Chemistry	6	196	32.67	9.8	5	Q1	England
10	Food and Chemical Toxicology	5	179	35.80	3.5	5	Q2	England

Table S5 Top 10 co-cited journals on NPs treatment of HUA from 2004 to 2026

Rank	Journal	citations	H-index (%)	IF (2026)	JCR division	Country
1	Journal of Ethnopharmacology	1144	25	5.4	Q1	Ireland
2	Journal of Agricultural and Food Chemistry	629	10	6.2	Q1	United States
3	Food Chemistry	503	5	9.8	Q1	England
4	Molecules	466	9	4.6	Q2	Switzerland
5	Frontiers in Pharmacology	414	14	4.8	Q1	Switzerland
6	Phytomedicine	355	11	8.3	Q1	Germany
7	Biomedicine & Pharmacotherapy	319	9	7.5	Q1	France
8	Food & Function	319	3	5.4	Q1	England
9	PLoS One	302	3	2.6	Q2	United States
10	Nutrients	264	4	5.0	Q1	Switzerland

Table S6 Top 10 most co-cited articles on NPs for HUA treatment 2004 to 2026

Rank	Title	Journal	citations	Publication year	First author	JCR	IF (2026)
1	Regulation of uric acid metabolism and excretion	International Journal of Cardiology	39	2016	Maiuolo, J	3.2	Q2
2	Effects of Biota orientalis extract and its flavonoid constituents, quercetin and rutin on serum uric acid levels in oxonate-induced mice and xanthine dehydrogenase and xanthine oxidase activities in mouse liver	Journal of Ethnopharmacology	39	2004	Zhu, JX	5.4	Q1
3	A Chinese herbal medicine Ermiao wan reduces serum uric acid level and inhibits liver xanthine dehydrogenase and xanthine oxidase in mice	Journal of Ethnopharmacology	33	2004	Kong, LD	5.4	Q1
4	Therapeutic effects of xanthine oxidase inhibitors: Renaissance half a century after the discovery of allopurinol	Pharmacological Reviews	33	2006	Pacher, P	17.3	Q1
5	Gout	Lancet	32	2021	Dalbeth, N	88.5	Q1
6	Curcumin attenuates potassium oxonate-induced hyperuricemia and kidney inflammation in mice	Biomedicine & Pharmacotherapy	30	2019	Chen, YE	7.5	Q1
7	Global epidemiology of gout: prevalence, incidence, treatment patterns and risk factors	Nature Reviews Rheumatology	29	2020	Dehlin, M	32.7	Q1
8	Decreased extra-renal urate excretion is a common cause of hyperuricemia	Nature Communications	28	2012	Takada, T	15.7	Q1
9	Novel Insights into the Inhibitory Mechanism of Kaempferol on Xanthine Oxidase	Journal of Agricultural and Food Chemistry	28	2015	Wang, YJ	6.2	Q1
10	Contemporary Prevalence of Gout and Hyperuricemia in the United States and Decadal Trends: The National Health and Nutrition Examination Survey, 2007-2016	Arthritis & Rheumatology	27	2019	Chen-Xu, M	20.9	Q1

Table S7 Top 10 high-frequency keywords for NPs treatments for HUA from 2004 to 2026

Rank	Keyword	Counts	Rank(Centrality)	Keyword	Counts	Centrality
1	uric acid	155	1	hyperuricemia	115	0.38
2	xanthine oxidase	153	2	gout	116	0.37
3	gout	116	3	extract	38	0.29
4	hyperuricemia	115	4	allopurinol	42	0.25
5	oxidative stress	85	5	epidemiology	12	0.17
6	serum uric acid	70	6	serum uric acid	70	0.15
7	inflammation	56	7	antioxidant activity	42	0.14
8	identification	43	8	potassium oxonate	15	0.14
9	allopurinol	42	9	flavonoids	35	0.12
10	antioxidant activity	42	10	activation	31	0.12

Table S8 Natural compounds for treating HUA

ID	Compound name	Structural formula	Category	Anti-hyperuricemie mechanism	Reference
1	Dihydroquercetin	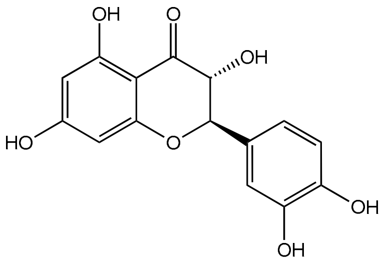	Flavonoid	Inhibit XOD activity	[76]
2	Quercetin	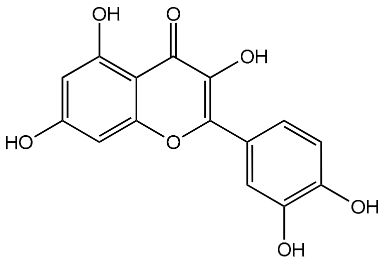	Flavonoid	Inhibit XOD activity	[76]
3	Biochanin A	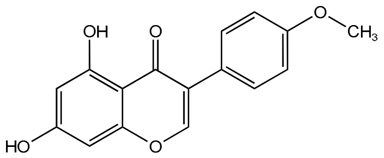	Flavonoid	Inhibit XOD activity	[76]

Continued Table S8 Natural compounds for treating HUA

ID	Compound name	Structural formula	Category	Anti-hyperuricemie mechanism	Reference

4	Epicatechin	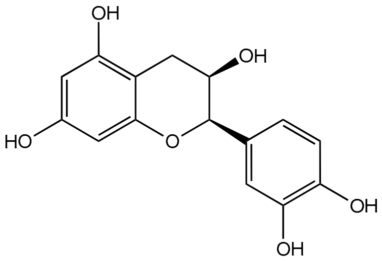	Flavonoid	Inhibit XOD activity	[76]
5	(2S) 5,2'-dihydroxy-6",6"-dimethylchromeno-(7,8:2",3")-flavanone	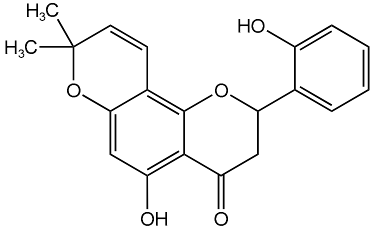	Flavonoid	Inhibit XOD activity	[77]
6	(2S) 5,2'-dihydroxy-6",6"-dimethylchromeno-(7,8:2",3")-3'-prenylflavanone	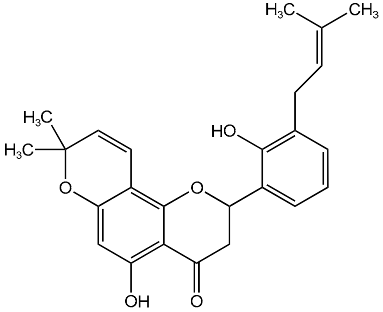	Flavonoid	Inhibit XOD activity	[77]

Continued Table S8 Natural compounds for treating HUA

ID	Compound name	Structural formula	Category	Anti-hyperuricemie mechanism	Reference

7	Obovatin	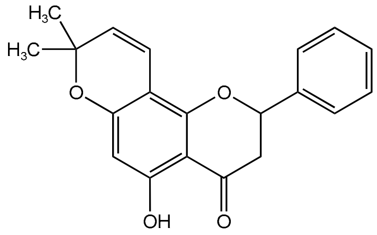	Flavonoid	Inhibit XOD activity	[77]
8	Kaempferol	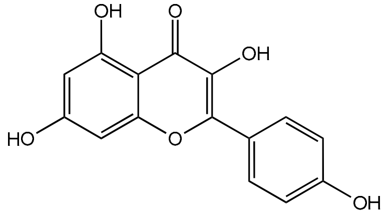	Flavonoid	Reduce uric acid levels	[79]
9	Iignan	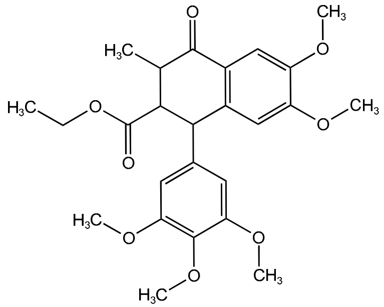	Flavonoid	Inhibit XOD activity	[78]

Continued Table S8 Natural compounds for treating HUA

ID	Compound name	Structural formula	Category	Anti-hyperuricemie mechanism	Reference

10	Amentoflavone	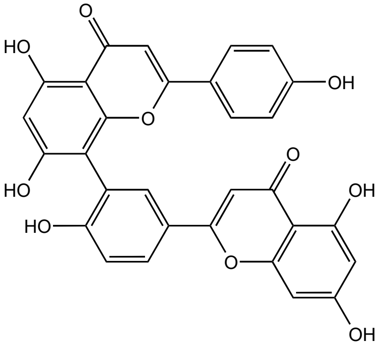	Flavonoid	Inhibit XOD activity	[78]
11	Cupressuflavone	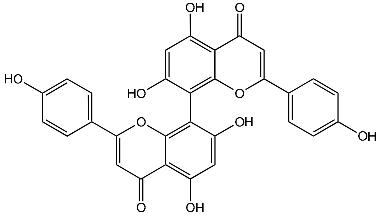	Flavonoid	Inhibit XOD activity	[78]
12	Isocryptomerin	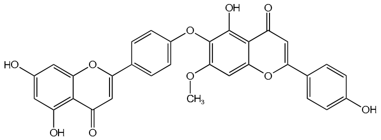	Flavonoid	Inhibit XOD activity	[78]
13	Hinokiflavone	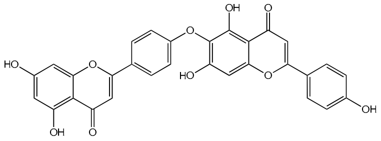	Flavonoid	Inhibit XOD activity	[78]

Continued Table S8 Natural compounds for treating HUA

ID	Compound name	Structural formula	Category	Anti-hyperuricemie mechanism	Reference

14	Neolignan	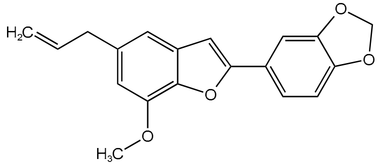	Flavonoid	Inhibit XOD activity	[78]
15	Luteolin	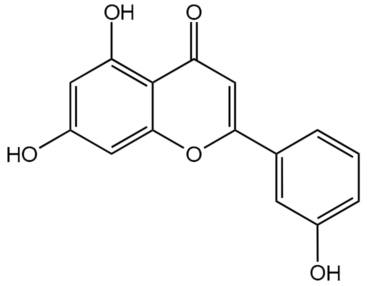	Flavonoid	Inhibit URAT1 activity	[81]
16	Naringenin	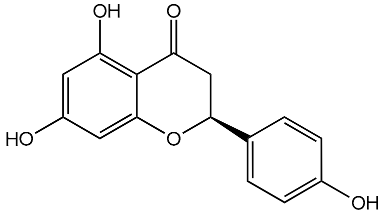	Flavonoid	Downregulating renal GLUT9 protein expression；upregulating renal ABCG2 protein expression; reducing levels of proinflammatory cytokines (IL-6, TNF-α)	[58]
17	kaempferol-7-O-β-D-glucopyranoside	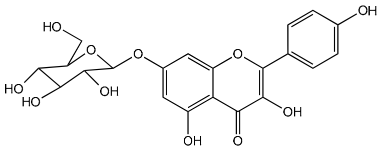	Flavonoid	Inhibit XOD activity；regulate the mRNA expression of renal urate transporters (URAT1,GLUT9,OAT1); downregulate the proinflammatory cytokines (TNF-α,IL-1β); reduce oxidative stress（MDA、NO、GSH、SOD）	[81]

Continued Table S8 Natural compounds for treating HUA

ID	Compound name	Structural formula	Category	Anti-hyperuricemie mechanism	Reference

18	kaempferol-3-O-β-D-glucopyranosyl (6→1)-α-L-rahmnopyranoside	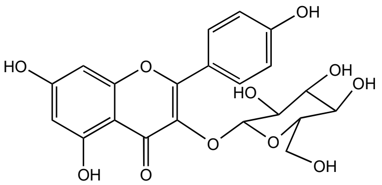	Flavonoid	Inhibit XOD activity；regulate the mRNA expression of renal urate transporters (URAT1,GLUT9,OAT1)；downregulate the proinflammatory cytokines (TNF-α,IL-1β)；Reduce oxidative stress（MDA、NO、GSH、SOD）	[81]
19	luteolin-3′,4′-di-O-β-D-glucopyranoside	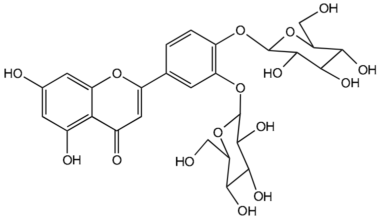	Flavonoid	Inhibit XOD activity；regulate the mRNA expression of renal urate transporters (URAT1,GLUT9,OAT1)；downregulate the proinflammatory cytokines (TNF-α,IL-1β)；Reduce oxidative stress（MDA、NO、GSH、SOD）	[81]
20	luteolin 7-O-β-D-glucopyranoside	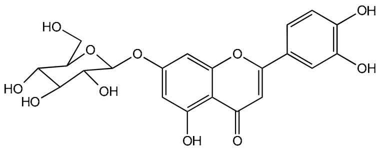	Flavonoid	Inhibit XOD activity；regulate the mRNA expression of renal urate transporters (URAT1,GLUT9,OAT1)；downregulate the proinflammatory cytokines (TNF-α,IL-1β)；Reduce oxidative stress（MDA、NO、GSH、SOD）	[81]

Continued Table S8 Natural compounds for treating HUA

ID	Compound name	Structural formula	Category	Anti-hyperuricemie mechanism	Reference

21	chrysoeriol	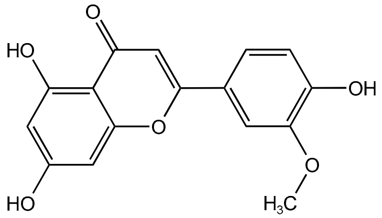	Flavonoid	Inhibit XOD activity；regulate the mRNA expression of renal urate transporters (URAT1,GLUT9,OAT1)；downregulate the proinflammatory cytokines (TNF-α,IL-1β)；Reduce oxidative stress（MDA、NO、GSH、SOD）	[81]
22	Apigenin	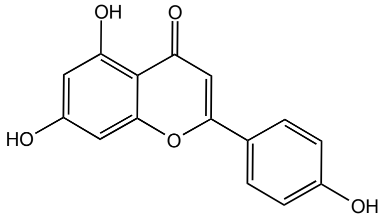	Flavonoid	Reduce uric acid levels	[83]
23	Fisetin	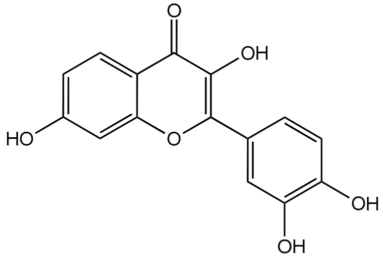	Flavonoid	Inhibit URAT1 activity	[80]

Continued Table S8 Natural compounds for treating HUA

ID	Compound name	Structural formula	Category	Anti-hyperuricemie mechanism	Reference

24	Gossypetin	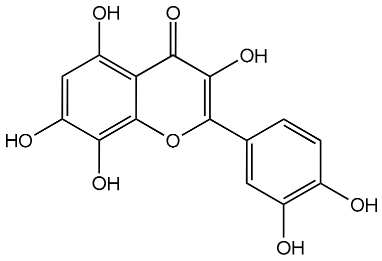	Flavonoid	Inhibit URAT1 activity	[80]
25	Morin	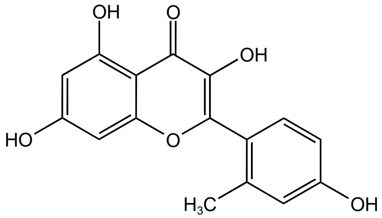	Flavonoid	Inhibit URAT1 activity	[80]
26	Myricetin	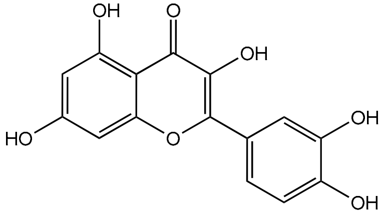	Flavonoid	Inhibit URAT1 activity	[80]

Continued Table S8 Natural compounds for treating HUA

ID	Compound name	Structural formula	Category	Anti-hyperuricemie mechanism	Reference

27	Quercetagetin	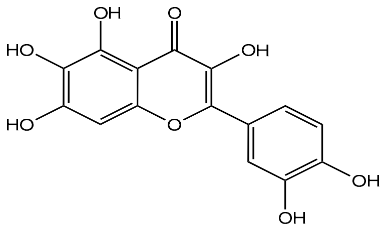	Flavonoid	Inhibit URAT1 activity	[80]
28	Genistein	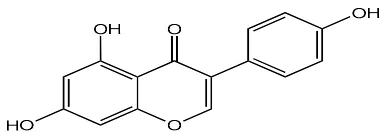	Flavonoid	Inhibit URAT1 activity	[80]
29	2'-Hydroxychalcone	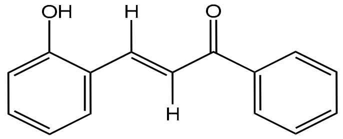	Flavonoid	Inhibit URAT1 activity	[80]
30	4-Hydroxychalcone	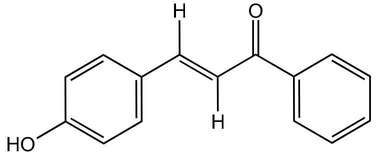	Flavonoid	Inhibit URAT1 activity	[80]
31	isoliquiritigenin	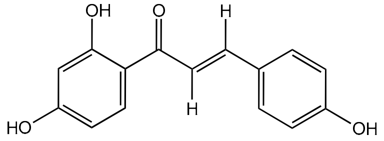	Flavonoid	Inhibit URAT1 activity	[80]

Continued Table S8 Natural compounds for treating HUA

ID	Compound name	Structural formula	Category	Anti-hyperuricemie mechanism	Reference

32	Naringenin chalcone	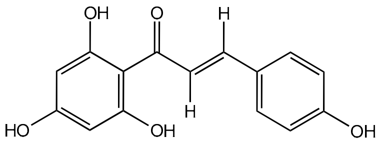	Flavonoid	Inhibit URAT1 activity	[80]
33	Phloretin	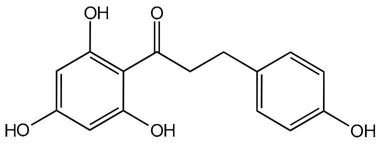	Flavonoid	Inhibit URAT1 activity	[80]
34	(2S)-5,7-dimethoxy-8,4'-dihydroxyflavanone	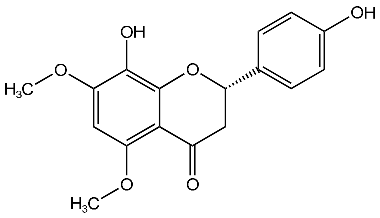	Flavonoid	Inhibit XOD activity	[57]
35	2',4'-dimethoxy-4,5',6'-trihydroxychalcone	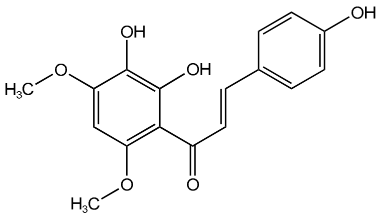	Flavonoid	Inhibit XOD activity	[57]

Continued Table S8 Natural compounds for treating HUA

ID	Compound name	Structural formula	Category	Anti-hyperuricemie mechanism	Reference

36	(Z)-4,6-dimethoxy-7,4'-dihydroxyaurone	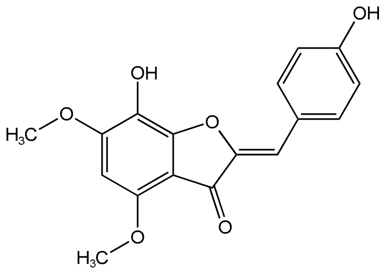	Flavonoid	Inhibit XOD activity	[57]
37	Ferulic acid	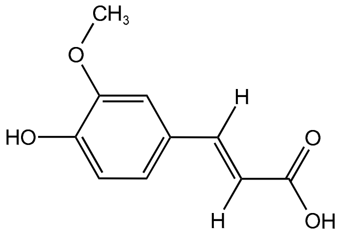	phenolic acid	Inhibit XOD activity	[85]
38	chlorogenic acid	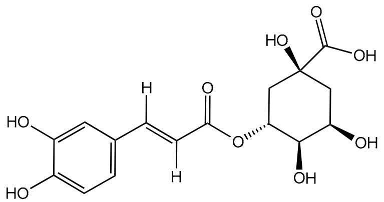	phenolic acid	Inhibit XOD activity	[86]
39	caffeic acid	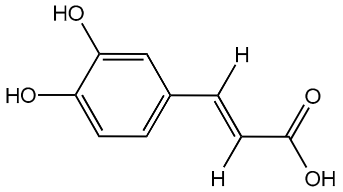	phenolic acid	Inhibit XOD activity	[86]

Continued Table S8 Natural compounds for treating HUA

ID	Compound name	Structural formula	Category	Anti-hyperuricemie mechanism	Reference

40	p-coumaric acid	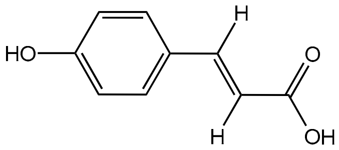	phenolic acid	Inhibit XOD activity	[86]
41	protocatechuic acid	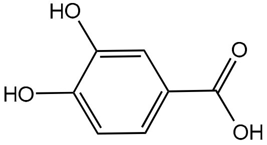	phenolic acid	Inhibit XOD activity	[86]
42	neochlorogenic acid	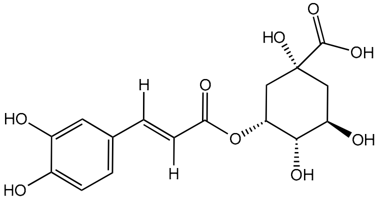	phenolic acid	Reduce serum uric acid levels；inhibition of renal cell apoptosis（Bax、caspase-3）;inhibit the inflammatory response(IL-1β, IL-18)	[87]
43	cryptochlorogenic acid	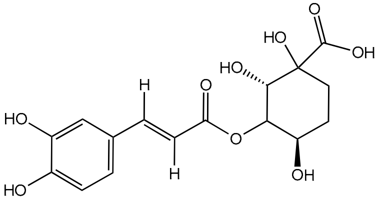	phenolic acid	Reduce serum uric acid levels；inhibition of renal cell apoptosis（Bax、caspase-3）;inhibit the inflammatory response(IL-1β, IL-18)	[87]
44	isochlorogenic acid A	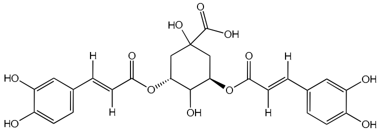	phenolic acid	Reduce serum uric acid levels；inhibition of renal cell apoptosis（Bax、caspase-3）;inhibit the inflammatory response(IL-1β, IL-18)	[87]

Continued Table S8 Natural compounds for treating HUA

ID	Compound name	Structural formula	Category	Anti-hyperuricemie mechanism	Reference

45	isochlorogenic acid B	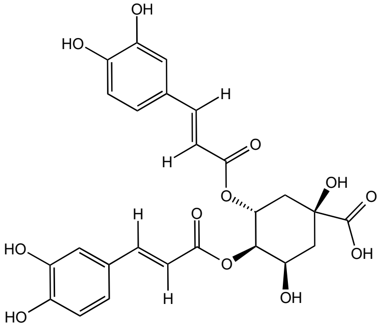	phenolic acid	Reduce serum uric acid levels；inhibition of renal cell apoptosis （Bax、caspase-3）;inhibit the inflammatory response(IL-1β, IL-18)	[87]
46	isochlorogenic acid C	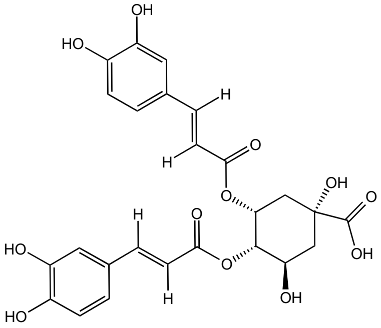	phenolic acid	Reduce serum uric acid levels；inhibition of renal cell apoptosis （Bax、caspase-3）;inhibit the inflammatory response(IL-1β, IL-18)	[87]
47	ellagic acid	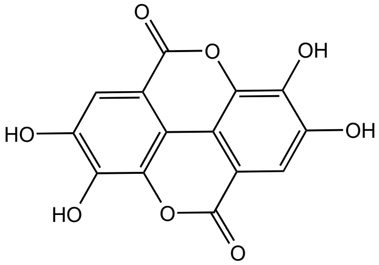	phenolic acid	Inhibit XOD activity;inhibition of oxidative stress (SOD, MDA);inhibit the inflammatory response（IL-1β、IL-6、TNF-α）;downregulate renal URAT1 protein expression;upregulate renal OAT1 and ABCG2 protein expression	[88]

Continued Table S8 Natural compounds for treating HUA

ID	Compound name	Structural formula	Category	Anti-hyperuricemie mechanism	Reference

48	Gallic acid	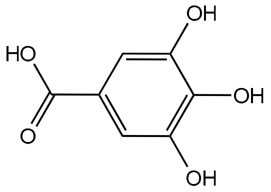	phenolic acid	Inhibit XOD activity;inhibition of oxidative stress (SOD, MDA);inhibit the inflammatory response（IL-1β、IL-6、TNF-α）;downregulate renal URAT1 protein expression;upregulate renal OAT1 and ABCG2 protein expression	[88]
49	Rosmarinic acid	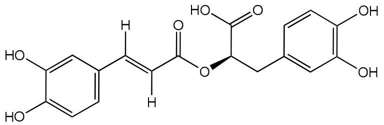	phenolic acid	Inhibit XOD activity	[89]
50	Chelerythrine	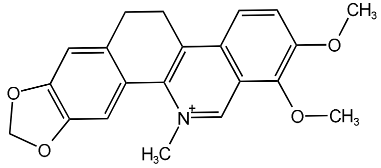	Alkaloid	Inhibit XDH activity	[91]
51	Palmatine	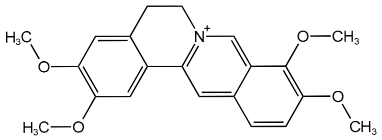	Alkaloid	Inhibit XOD and ADA activities；downregulate renal protein expression of URAT1, GLUT9; upregulate the renal protein expression of ABCG2 and OTA1;activate the Keap1-Nrf2 pathway; inhibit the TXNIP/NLRP3 inflammasome pathway (caspase-1, IL-1β, IL-18)	[92]

Continued Table S8 Natural compounds for treating HUA

ID	Compound name	Structural formula	Category	Anti-hyperuricemie mechanism	Reference

52	Nuciferine	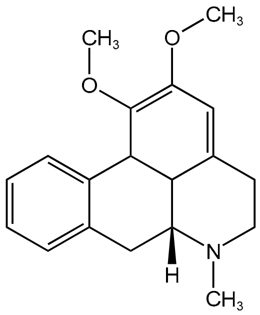	Alkaloid	Inhibit the TLR4/NF-κB pathway;downregulate renal protein expression of URAT1, GLUT9	[93]
53	Piperine	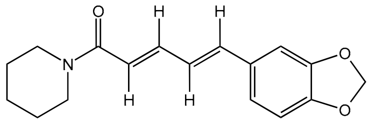	Alkaloid	Inhibit the AKT/mTOR pathway;downregulate renal protein expression of URAT1, GLUT9	[94]
54	Berberine	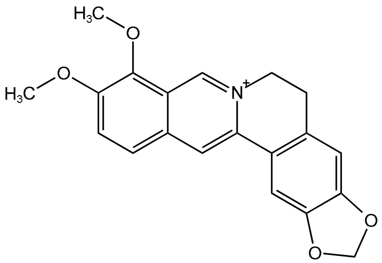	Alkaloid	Inhibit XOD activity; regulate inflammatory and apoptotic pathways	[95]

Continued Table S8 Natural compounds for treating HUA

ID	Compound name	Structural formula	Category	Anti-hyperuricemie mechanism	Reference

55	Emodin	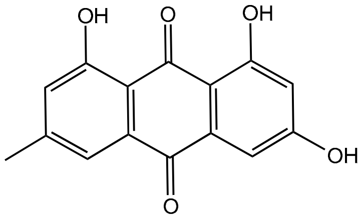	Quinone	Increase fractional excretion of uric acid (FEUA)	[96]
56	Rhein	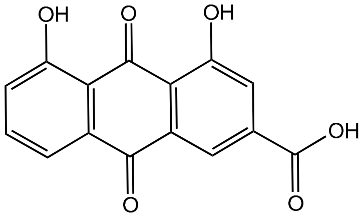	Quinone	Inhibit XOD activity; reduce the production of proinflammatory cytokines(IL-1β, PGE-2, TNF-α);inhibit the expression（TGF-β1）	[97]
57	juglone	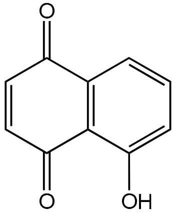	Quinone	Inhibit XOD activity	[98]
58	menadione	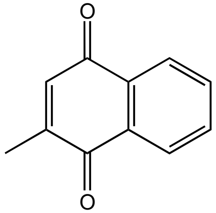	Quinone	Inhibit XOD activity	[98]

Continued Table S8 Natural compounds for treating HUA

ID	Compound name	Structural formula	Category	Anti-hyperuricemie mechanism	Reference

59	9,10-phenanthrenequinone	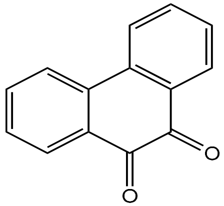	Quinone	Inhibit XOD activity	[98]
60	quinalizarin	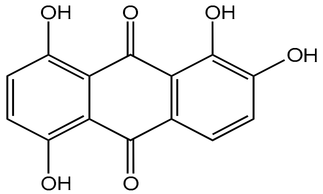	Quinone	Inhibit XOD activity	[98]
61	N-(9,10-anthraquinone-2-ylcarbonyl)	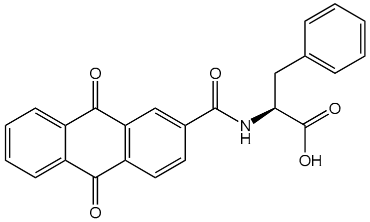	Quinone	Inhibit XOD activity; downregulate renal GLUT9； upregulate renal OAT1/OAT3 protein levels; inhibit the NLRP3 inflammatory pathway (TNF-α, IL-6, IL-1β)	[99]
62	Lanosteryl Triterpene	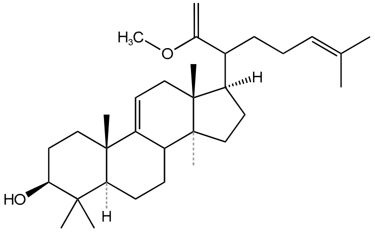	Terpene	Reduce serum uric acid levels	[100]
