# Supplementary material for: Global Trends and Research Hotspots in Natural Products for Hyperuricemia: A Bibliometric Analysis (2004–2026)
Source: Pharmaceuticals (Basel). 2026 Aug 14;19(8):1286. doi: 10.3390/ph19081286 (PMC13517059; doi:10.3390/ph19081286)
Supplement: Supplementary file 1 [file pharmaceuticals-19-01286-s001.zip › File S1-Supplementary Methods Database Specific Search Strings.pdf]

Supplementary Methods: Database-Specific Search Strings

This supplementary document provides the complete, database-specific search strings and filtering procedures used for literature retrieval in PubMed, Scopus, and Web of Science. The filtering procedures were adapted to each database's native interface: PubMed and Scopus do not support month/day-level date filtering, so date, document type, and language filters were embedded directly within the search query in four progressive steps. In contrast, Web of Science supports precise YYYY-MM-DD date filtering via its built-in Publication Date sidebar filter, so the date filter was applied via the sidebar interface while document type and language filters were embedded within the search query. The number of records retrieved at each step is reported to ensure full transparency and reproducibility.

1. Web of Science Core Collection

Database: Web of Science Core Collection (WoSCC)

Search field: Topic (TS = title, abstract, author keywords, and Keywords Plus)

Search date: April 1, 2026

Filtering strategy: Web of Science is the only database among the three that supports precise YYYY-MM-DD date filtering via its built-in "Publication Date" sidebar refinement option. To leverage this capability and ensure the most accurate date coverage, the date range filter (2004-01-01 to 2026-04-01) was applied via the sidebar interface (Step 2 below). The document type and language filters were then embedded directly within the search query (Steps 3 and 4). The search was executed in four progressive cumulative steps.

Step 1.1 Keyword search (no filter)

Filter applied: None (keyword combination only)

Query:

(TS=("Traditional Chinese Medicine" OR "Chinese Medicine" OR "Chinese Herb*" OR "Herbal Medicine" OR "Herb* Extract*" OR "Phytochemical*" OR "Phytotherap*" OR "Natural Product*" OR "Natural Compound*" OR "Bioactive Compound*" OR "Plant Extract*" OR "Medicinal Plant*" OR "Plant-Derived*" OR "Secondary Metabolit*" OR "Botanical Medicine" OR "Ethnopharmacology" OR "Ethnomedicine" OR "Herbal Formulation*" OR "Natural Medicine*" OR "Pharmacological Activit*" OR "Plant-Based Medicine") AND TS=("Hyperuricemia*" OR "Hyperuricaemia*" OR "High Uric Acid" OR "Elevated Uric Acid" OR "Serum Uric Acid" OR "Urate Metabolism" OR "Uric Acid Metabolism" OR "Uric Acid Homeostasis" OR "Urate-Lowering Therap*" OR "Renal Uric Acid Excretion"))

Records retrieved: 726

Step 1.2 + Date range filter (via sidebar)

Filter applied: Publication Date (sidebar refinement): from 2004-01-01 to 2026-04-01 (precise YYYY-MM-DD format supported by WOS)

Query (same as Step 3.1; date filter applied via WOS sidebar "Publication Date" option, not within the query):

(TS=("Traditional Chinese Medicine" OR "Chinese Medicine" OR "Chinese Herb*" OR "Herbal Medicine" OR "Herb* Extract*" OR "Phytochemical*" OR "Phytotherap*" OR "Natural Product*" OR "Natural Compound*" OR "Bioactive Compound*" OR "Plant Extract*" OR

"Medicinal Plant*" OR "Plant-Derived*" OR "Secondary Metabolit*" OR "Botanical Medicine" OR "Ethnopharmacology" OR "Ethnomedicine" OR "Herbal Formulation*" OR "Natural Medicine*" OR "Pharmacological Activit*" OR "Plant-Based Medicine") AND TS=("Hyperuricemia*" OR "Hyperuricaemia*" OR "High Uric Acid" OR "Elevated Uric Acid" OR "Serum Uric Acid" OR "Urate Metabolism" OR "Uric Acid Metabolism" OR "Uric Acid Homeostasis" OR "Urate-Lowering Therap*" OR "Renal Uric Acid Excretion"))

Records retrieved: 700

Step 1.3 + Document type filter

Filter applied: Document Type: Article and Review (embedded in query as DT field)

Query (date filter remains applied via sidebar; document type filter added to query):

(TS=("Traditional Chinese Medicine" OR "Chinese Medicine" OR "Chinese Herb*" OR "Herbal Medicine" OR "Herb* Extract*" OR "Phytochemical*" OR "Phytotherap*" OR "Natural Product*" OR "Natural Compound*" OR "Bioactive Compound*" OR "Plant Extract*" OR "Medicinal Plant*" OR "Plant-Derived*" OR "Secondary Metabolit*" OR "Botanical Medicine" OR "Ethnopharmacology" OR "Ethnomedicine" OR "Herbal Formulation*" OR "Natural Medicine*" OR "Pharmacological Activit*" OR "Plant-Based Medicine") AND TS=("Hyperuricemia*" OR "Hyperuricaemia*" OR "High Uric Acid" OR "Elevated Uric Acid" OR "Serum Uric Acid" OR "Urate Metabolism" OR "Uric Acid Metabolism" OR "Uric Acid Homeostasis" OR "Urate-Lowering Therap*" OR "Renal Uric Acid Excretion")) AND DT=(Article OR Review)

Records retrieved: 694

Step 1.4 + Language filter

Filter applied: Language: English (embedded in query as LA field)

Query (date filter via sidebar; document type and language filters embedded in query):

(TS=("Traditional Chinese Medicine" OR "Chinese Medicine" OR "Chinese Herb*" OR "Herbal Medicine" OR "Herb* Extract*" OR "Phytochemical*" OR "Phytotherap*" OR "Natural Product*" OR "Natural Compound*" OR "Bioactive Compound*" OR "Plant Extract*" OR "Medicinal Plant*" OR "Plant-Derived*" OR "Secondary Metabolit*" OR "Botanical Medicine" OR "Ethnopharmacology" OR "Ethnomedicine" OR "Herbal Formulation*" OR "Natural Medicine*" OR "Pharmacological Activit*" OR "Plant-Based Medicine") AND TS=("Hyperuricemia*" OR "Hyperuricaemia*" OR "High Uric Acid" OR "Elevated Uric Acid" OR "Serum Uric Acid" OR "Urate Metabolism" OR "Uric Acid Metabolism" OR "Uric Acid Homeostasis" OR "Urate-Lowering Therap*" OR "Renal Uric Acid Excretion")) AND DT=(Article OR Review) AND LA=English

Records retrieved: 690 (final dataset for export)

2. PubMed

Search field: Title/Abstract and MeSH Terms

Search date: April 1, 2026

Filtering strategy: PubMed does not support precise day/month-level date filtering. All filters (date range, document type, language) were therefore embedded directly within the search query

using field tags, and the search was executed in four progressive cumulative steps.

Step 2.1 Keyword search (no filter)
Filter applied: None (keyword combination only)
Query:
("Medicine, Chinese Traditional"[MeSH Terms] OR "Traditional Chinese Medicine"[Title/Abstract] OR "Chinese Medicine"[Title/Abstract] OR "Chinese Herb*"[Title/Abstract] OR "Herbal Medicine"[MeSH Terms] OR "Herb* Extract*"[Title/Abstract] OR "Phytochemical*"[Title/Abstract] OR "Phytotherap*"[Title/Abstract] OR "Natural Product*"[Title/Abstract] OR "Natural Compound*"[Title/Abstract] OR "Bioactive Compound*"[Title/Abstract] OR "Plant Extracts"[MeSH Terms] OR "Plants, Medicinal"[MeSH Terms] OR "Plant-Derived*"[Title/Abstract] OR "Secondary Metabolit*"[Title/Abstract] OR "Botanical Medicine"[Title/Abstract] OR "Ethnopharmacology"[MeSH Terms] OR "Ethnomedicine"[Title/Abstract] OR "Herbal Formulation*"[Title/Abstract] OR "Natural Medicine*"[Title/Abstract] OR "Pharmacological Activit*"[Title/Abstract] OR "Plant-Based Medicine"[Title/Abstract]) AND ("Hyperuricemia"[MeSH Terms] OR "Hyperuricemia*"[Title/Abstract] OR "High Uric Acid"[Title/Abstract] OR "Elevated Uric Acid"[Title/Abstract] OR "Serum Uric Acid"[Title/Abstract] OR "Urate Metabolism"[Title/Abstract] OR "Uric Acid Metabolism"[Title/Abstract] OR "Uric Acid Homeostasis"[Title/Abstract] OR "Urate-Lowering Therap*"[Title/Abstract] OR "Renal Uric Acid Excretion"[Title/Abstract])
Records retrieved: 853

Step 2.2 + Date range filter
Filter applied: Publication date: 2004/01/01 to 2026/04/01 (year/month/day-level precision available in PubMed)
Query:
("Medicine, Chinese Traditional"[MeSH Terms] OR "Traditional Chinese Medicine"[Title/Abstract] OR "Chinese Medicine"[Title/Abstract] OR "Chinese Herb*"[Title/Abstract] OR "Herbal Medicine"[MeSH Terms] OR "Herb* Extract*"[Title/Abstract] OR "Phytochemical*"[Title/Abstract] OR "Phytotherap*"[Title/Abstract] OR "Natural Product*"[Title/Abstract] OR "Natural Compound*"[Title/Abstract] OR "Bioactive Compound*"[Title/Abstract] OR "Plant Extracts"[MeSH Terms] OR "Plants, Medicinal"[MeSH Terms] OR "Plant-Derived*"[Title/Abstract] OR "Secondary Metabolit*"[Title/Abstract] OR "Botanical Medicine"[Title/Abstract] OR "Ethnopharmacology"[MeSH Terms] OR "Ethnomedicine"[Title/Abstract] OR "Herbal Formulation*"[Title/Abstract] OR "Natural Medicine*"[Title/Abstract] OR "Pharmacological Activit*"[Title/Abstract] OR "Plant-Based Medicine"[Title/Abstract]) AND ("Hyperuricemia"[MeSH Terms] OR "Hyperuricemia*"[Title/Abstract] OR "High Uric Acid"[Title/Abstract] OR "Elevated Uric Acid"[Title/Abstract] OR "Serum Uric Acid"[Title/Abstract] OR "Urate Metabolism"[Title/Abstract] OR "Uric Acid Metabolism"[Title/Abstract] OR "Uric Acid Homeostasis"[Title/Abstract] OR "Urate-Lowering Therap*"[Title/Abstract] OR "Renal Uric Acid Excretion"[Title/Abstract]) AND ("2004/01/01"[Date - Publication] : "2026/04/01"[Date - Publication])

Records retrieved: 814

Step 2.3 + Document type filter

Filter applied: Publication type: Journal Article [ptyp] OR Review [ptyp]

Query:

("Medicine, Chinese Traditional"[MeSH Terms] OR "Traditional Chinese Medicine"[Title/Abstract] OR "Chinese Medicine"[Title/Abstract] OR "Chinese Herb*"[Title/Abstract] OR "Herbal Medicine"[MeSH Terms] OR "Herb* Extract*"[Title/Abstract] OR "Phytochemical*"[Title/Abstract] OR "Phytotherap*"[Title/Abstract] OR "Natural Product*"[Title/Abstract] OR "Natural Compound*"[Title/Abstract] OR "Bioactive Compound*"[Title/Abstract] OR "Plant Extracts"[MeSH Terms] OR "Plants, Medicinal"[MeSH Terms] OR "Plant-Derived*"[Title/Abstract] OR "Secondary Metabolit*"[Title/Abstract] OR "Botanical Medicine"[Title/Abstract] OR "Ethnopharmacology"[MeSH Terms] OR "Ethnomedicine"[Title/Abstract] OR "Herbal Formulation*"[Title/Abstract] OR "Natural Medicine*"[Title/Abstract] OR "Pharmacological Activit*"[Title/Abstract] OR "Plant-Based Medicine"[Title/Abstract]) AND ("Hyperuricemia"[MeSH Terms] OR "Hyperuricemia*"[Title/Abstract] OR "High Uric Acid"[Title/Abstract] OR "Elevated Uric Acid"[Title/Abstract] OR "Serum Uric Acid"[Title/Abstract] OR "Urate Metabolism"[Title/Abstract] OR "Uric Acid Metabolism"[Title/Abstract] OR "Uric Acid Homeostasis"[Title/Abstract] OR "Urate-Lowering Therap*"[Title/Abstract] OR "Renal Uric Acid Excretion"[Title/Abstract]) AND ("2004/01/01"[Date - Publication] : "2026/04/01"[Date - Publication]) AND (Journal Article[ptyp] OR Review[ptyp])

Records retrieved: 811

Step 2.4 + Language filter

Filter applied: Language: English [la]

Query:

("Medicine, Chinese Traditional"[MeSH Terms] OR "Traditional Chinese Medicine"[Title/Abstract] OR "Chinese Medicine"[Title/Abstract] OR "Chinese Herb*"[Title/Abstract] OR "Herbal Medicine"[MeSH Terms] OR "Herb* Extract*"[Title/Abstract] OR "Phytochemical*"[Title/Abstract] OR "Phytotherap*"[Title/Abstract] OR "Natural Product*"[Title/Abstract] OR "Natural Compound*"[Title/Abstract] OR "Bioactive Compound*"[Title/Abstract] OR "Plant Extracts"[MeSH Terms] OR "Plants, Medicinal"[MeSH Terms] OR "Plant-Derived*"[Title/Abstract] OR "Secondary Metabolit*"[Title/Abstract] OR "Botanical Medicine"[Title/Abstract] OR "Ethnopharmacology"[MeSH Terms] OR "Ethnomedicine"[Title/Abstract] OR "Herbal Formulation*"[Title/Abstract] OR "Natural Medicine*"[Title/Abstract] OR "Pharmacological Activit*"[Title/Abstract] OR "Plant-Based Medicine"[Title/Abstract]) AND ("Hyperuricemia"[MeSH Terms] OR "Hyperuricemia*"[Title/Abstract] OR "High Uric Acid"[Title/Abstract] OR "Elevated Uric Acid"[Title/Abstract] OR "Serum Uric Acid"[Title/Abstract] OR "Urate Metabolism"[Title/Abstract] OR "Uric Acid Metabolism"[Title/Abstract] OR "Uric Acid Homeostasis"[Title/Abstract] OR "Urate-Lowering Therap*"[Title/Abstract] OR "Renal Uric Acid Excretion"[Title/Abstract]) AND ("2004/01/01"[Date - Publication] : "2026/04/01"[Date - Publication]) AND (Journal Article[ptyp] OR Review[ptyp]) AND English[la]

Records retrieved: 763 (final dataset for export)

3. Scopus
Search field: TITLE-ABS-KEY (title, abstract, and keywords)
Search date: April 1, 2026
Filtering strategy: Scopus supports year-level date filtering only (PUBYEAR); month/day-level precision is not available. All filters (year, document type, language) were therefore embedded directly within the search query, and the search was executed in four progressive cumulative steps.

Step 3.1 Keyword search (no filter)
Filter applied: None (keyword combination only)
Query:
TITLE-ABS-KEY("Traditional Chinese Medicine" OR "Chinese Medicine" OR "Chinese Herb*" OR "Herbal Medicine" OR "Herb* Extract*" OR "Phytochemical*" OR "Phytotherap*" OR "Natural Product*" OR "Natural Compound*" OR "Bioactive Compound*" OR "Plant Extract*" OR "Medicinal Plant*" OR "Plant-Derived*" OR "Secondary Metabolit*" OR "Botanical Medicine" OR "Ethnopharmacology" OR "Ethnomedicine" OR "Herbal Formulation*" OR "Natural Medicine*" OR "Pharmacological Activit*" OR "Plant-Based Medicine") AND TITLE-ABS-KEY("Hyperuricemia*" OR "Hyperuricaemia*" OR "High Uric Acid" OR "Elevated Uric Acid" OR "Serum Uric Acid" OR "Urate Metabolism" OR "Uric Acid Metabolism" OR "Uric Acid Homeostasis" OR "Urate-Lowering Therap*" OR "Renal Uric Acid Excretion")
Records retrieved: 1481

Step 3.2 + Publication year filter
Filter applied: Publication year: 2004 to 2026 (year-level precision only)
Query:
TITLE-ABS-KEY("Traditional Chinese Medicine" OR "Chinese Medicine" OR "Chinese Herb*" OR "Herbal Medicine" OR "Herb* Extract*" OR "Phytochemical*" OR "Phytotherap*" OR "Natural Product*" OR "Natural Compound*" OR "Bioactive Compound*" OR "Plant Extract*" OR "Medicinal Plant*" OR "Plant-Derived*" OR "Secondary Metabolit*" OR "Botanical Medicine" OR "Ethnopharmacology" OR "Ethnomedicine" OR "Herbal Formulation*" OR "Natural Medicine*" OR "Pharmacological Activit*" OR "Plant-Based Medicine") AND TITLE-ABS-KEY("Hyperuricemia*" OR "Hyperuricaemia*" OR "High Uric Acid" OR "Elevated Uric Acid" OR "Serum Uric Acid" OR "Urate Metabolism" OR "Uric Acid Metabolism" OR "Uric Acid Homeostasis" OR "Urate-Lowering Therap*" OR "Renal Uric Acid Excretion") AND PUBYEAR > 2003 AND PUBYEAR < 2027
Records retrieved: 1315

Step 3.3 + Document type filter
Filter applied: Document type: Article (ar) and Review (re)
Query:
TITLE-ABS-KEY("Traditional Chinese Medicine" OR "Chinese Medicine" OR "Chinese Herb*" OR "Herbal Medicine" OR "Herb* Extract*" OR "Phytochemical*" OR "Phytotherap*" OR

"Natural Product*" OR "Natural Compound*" OR "Bioactive Compound*" OR "Plant Extract*" OR "Medicinal Plant*" OR "Plant-Derived*" OR "Secondary Metabolit*" OR "Botanical Medicine" OR "Ethnopharmacology" OR "Ethnomedicine" OR "Herbal Formulation*" OR "Natural Medicine*" OR "Pharmacological Activit*" OR "Plant-Based Medicine") AND TITLE-ABS-KEY("Hyperuricemia*" OR "Hyperuricaemia*" OR "High Uric Acid" OR "Elevated Uric Acid" OR "Serum Uric Acid" OR "Urate Metabolism" OR "Uric Acid Metabolism" OR "Uric Acid Homeostasis" OR "Urate-Lowering Therap*" OR "Renal Uric Acid Excretion") AND PUBYEAR > 2003 AND PUBYEAR < 2027 AND (LIMIT-TO(DOCTYPE,"ar") OR LIMIT-TO(DOCTYPE,"re"))
Records retrieved: 1276

Step 3.4 + Language filter
Filter applied: Language: English
Query:
TITLE-ABS-KEY("Traditional Chinese Medicine" OR "Chinese Medicine" OR "Chinese Herb*" OR "Herbal Medicine" OR "Herb* Extract*" OR "Phytochemical*" OR "Phytotherap*" OR "Natural Product*" OR "Natural Compound*" OR "Bioactive Compound*" OR "Plant Extract*" OR "Medicinal Plant*" OR "Plant-Derived*" OR "Secondary Metabolit*" OR "Botanical Medicine" OR "Ethnopharmacology" OR "Ethnomedicine" OR "Herbal Formulation*" OR "Natural Medicine*" OR "Pharmacological Activit*" OR "Plant-Based Medicine") AND TITLE-ABS-KEY("Hyperuricemia*" OR "Hyperuricaemia*" OR "High Uric Acid" OR "Elevated Uric Acid" OR "Serum Uric Acid" OR "Urate Metabolism" OR "Uric Acid Metabolism" OR "Uric Acid Homeostasis" OR "Urate-Lowering Therap*" OR "Renal Uric Acid Excretion") AND PUBYEAR > 2003 AND PUBYEAR < 2027 AND (LIMIT-TO(DOCTYPE,"ar") OR LIMIT-TO(DOCTYPE,"re")) AND (LIMIT-TO(LANGUAGE,"English"))
Records retrieved: 1141 (final dataset for export)

Notes:
1. In Web of Science (Section 1), the date range filter was applied via the built-in "Publication Date" sidebar refinement option (which supports precise YYYY-MM-DD date selection), while the document type and language filters were embedded within the search query. This approach leveraged WOS's unique capability for precise date filtering.
2. In PubMed (Section 2) and Scopus (Section 3), all filters (date/year, document type, language) were embedded directly within the search query using database-specific field tags, and the search was executed in four cumulative steps. Each query builds upon the previous one by appending an additional filter.
3. The differences in filtering approach across databases reflect the native interface limitations of each database: PubMed and Scopus do not support month/day-level date filtering (PubMed date field allows YYYY/MM/DD format; Scopus supports year-level only), whereas Web of Science supports precise YYYY-MM-DD date filtering via its sidebar.
